# Supplementary material for: Allosteric inhibition of PPM1D serine/threonine phosphatase via an altered conformational state
Source: Nat Commun. 2022 Jun 30;13:3778. doi: 10.1038/s41467-022-30463-9 (PMC9246869; doi:10.1038/s41467-022-30463-9)
Supplement: Supplementary file 1 — Supplementary Information [file 41467_2022_30463_MOESM1_ESM.pdf]

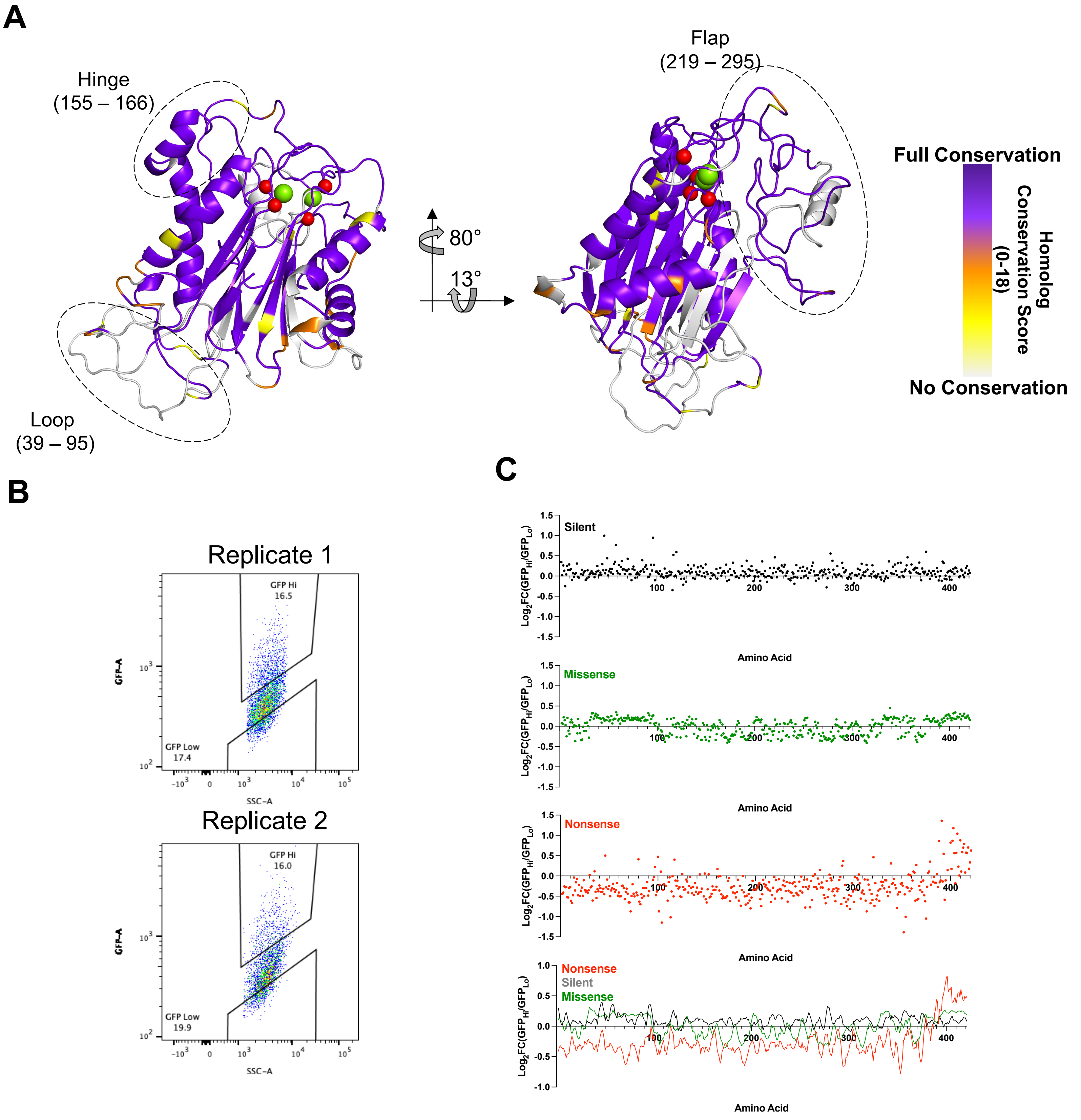

Supplementary Figure 1

(A) PPM1D ortholog values superimposed on homology model. For each residue, the degree of conservation across 19 PPM1D orthologs was assessed and scored from 0-19.

(B) Florescence-activated cell sorting (FACS) gating strategy used for saturation mutagenesis screen.

(C) Scores at each amino acid site derived from the saturation mutagenesis screen plotted for silent (black), missense (green), and nonsense (red) mutations. The missense mutation data reflects an average over all missense variants. A smooth curve fit for all three mutation types are shown at the bottom.

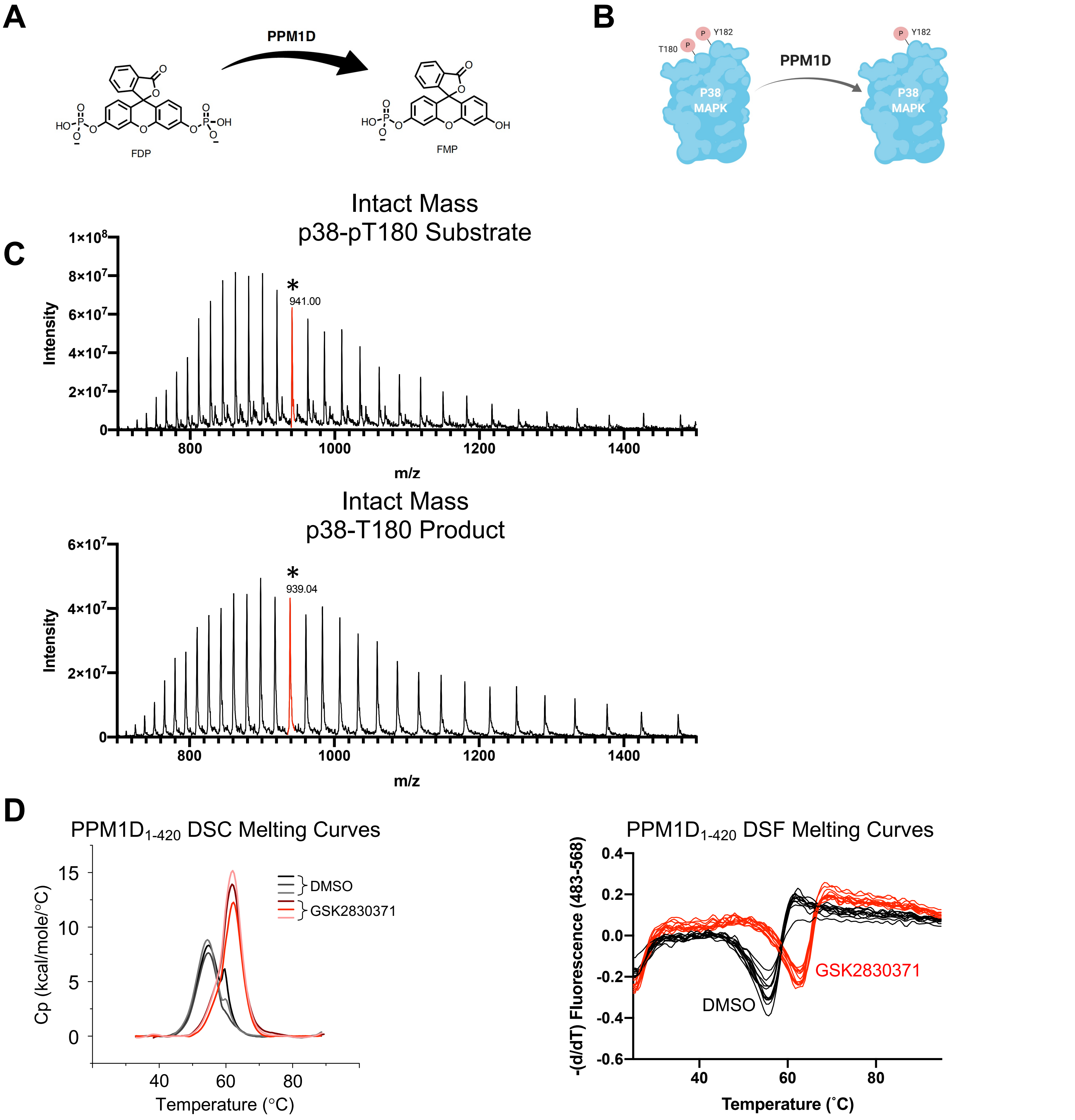

Supplementary Figure 2

(A) Schematic of FDP assay in which FDP is converted to FMP by recombinant PPM1D. The readout is by fluorescent detection.

(B) Schematic of P38 MAPK assay in which T180 is converted from its phosphorylated to its dephosphorylated form by PPM1D. The readout is by mass spectrometry.

(C) Intact mass spectrum of P38 MAPK in the phosphorylated (top) and dephosphorylated (bottom) states. Asterisks indicate the charge states selected for multiple-reaction-monitoring (MRM) analysis in the enzymatic assay. For more details, see Materials and Methods.

(D) Raw data for DSC (left) and DSF (right) shown in Figure 3C (PPM1D$_{1-420}$ in the absence (black) and presence (red) of GSK2830371).

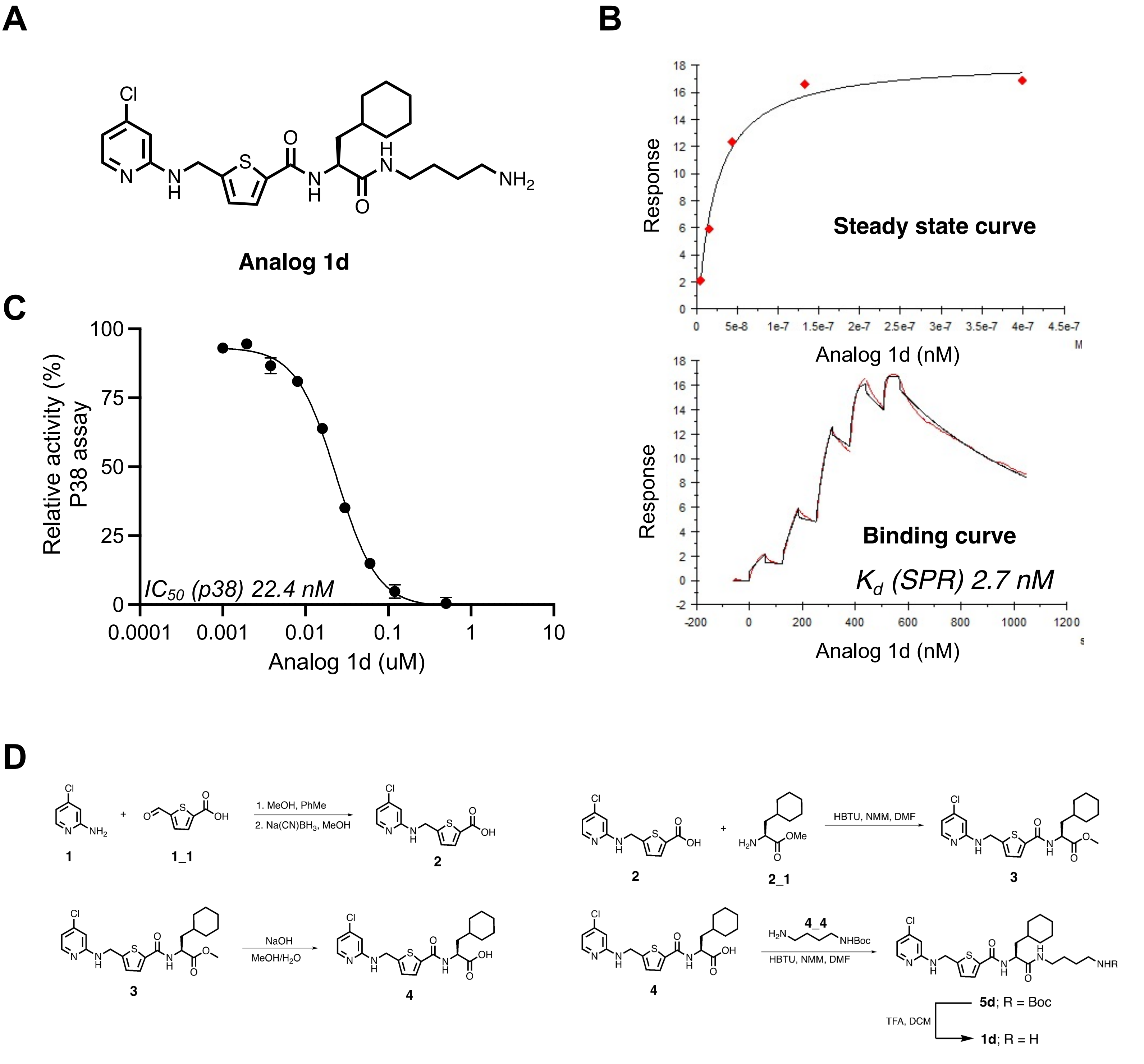

Supplementary Figure 3

(A) Structure of Analog 1d.

(B) SPR data for analog 1d (steady state curve on top, binding curve on bottom).

(C) Activity of analog 1d in P38 MAPK assay. N=4 independent sample runs. The horizontal bar represents the mean. Source data are provided as a Source Data file.

(D) Synthesis of analog 1d (see methods section for full details of synthesis).

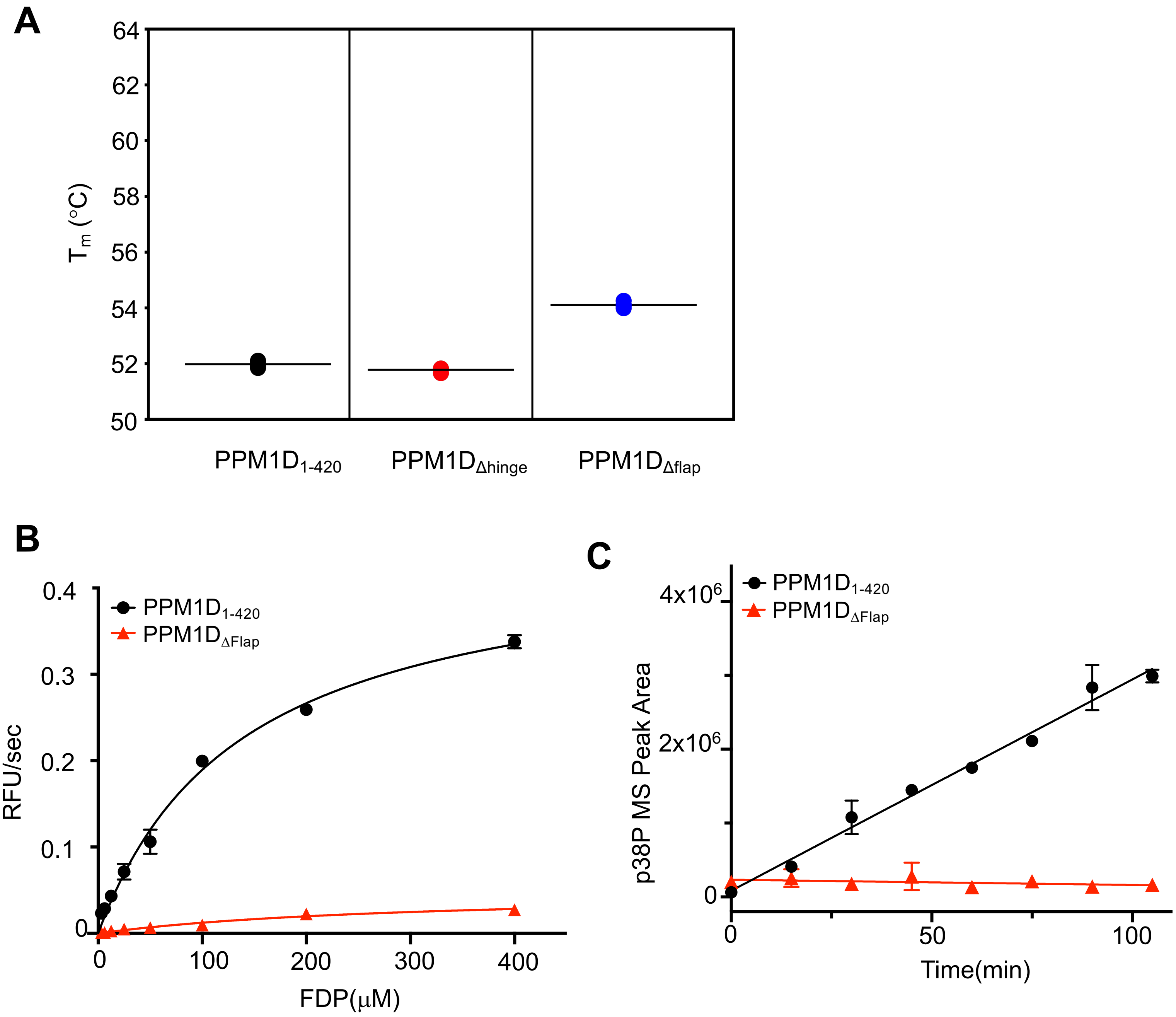

Supplementary Figure 4

(A) Differential scanning fluorimetry (DSF) showing the melting temperatures of PPM1D$_{1\text{-}420}$ (black) PPM1D$_{\Delta hinge}$ (red) and PPM1D$_{\Delta flap}$ (blue). N=4 independent sample runs. Data are presented as mean values +/- SD.

(B) Activity of PPM1D$_{1\text{-}420}$ and PPM1D$_{\Delta Flap}$ in the FDP assay. N=2 independent sample runs. Data are presented as mean values +/- SD.

(C) Activity of PPM1D$_{1\text{-}420}$ and PPM1D$_{\Delta Flap}$ in the P38 assay. N=3 independent samples runs. Data are presented as mean values +/- SD.

Source data are provided as a Source Data file.

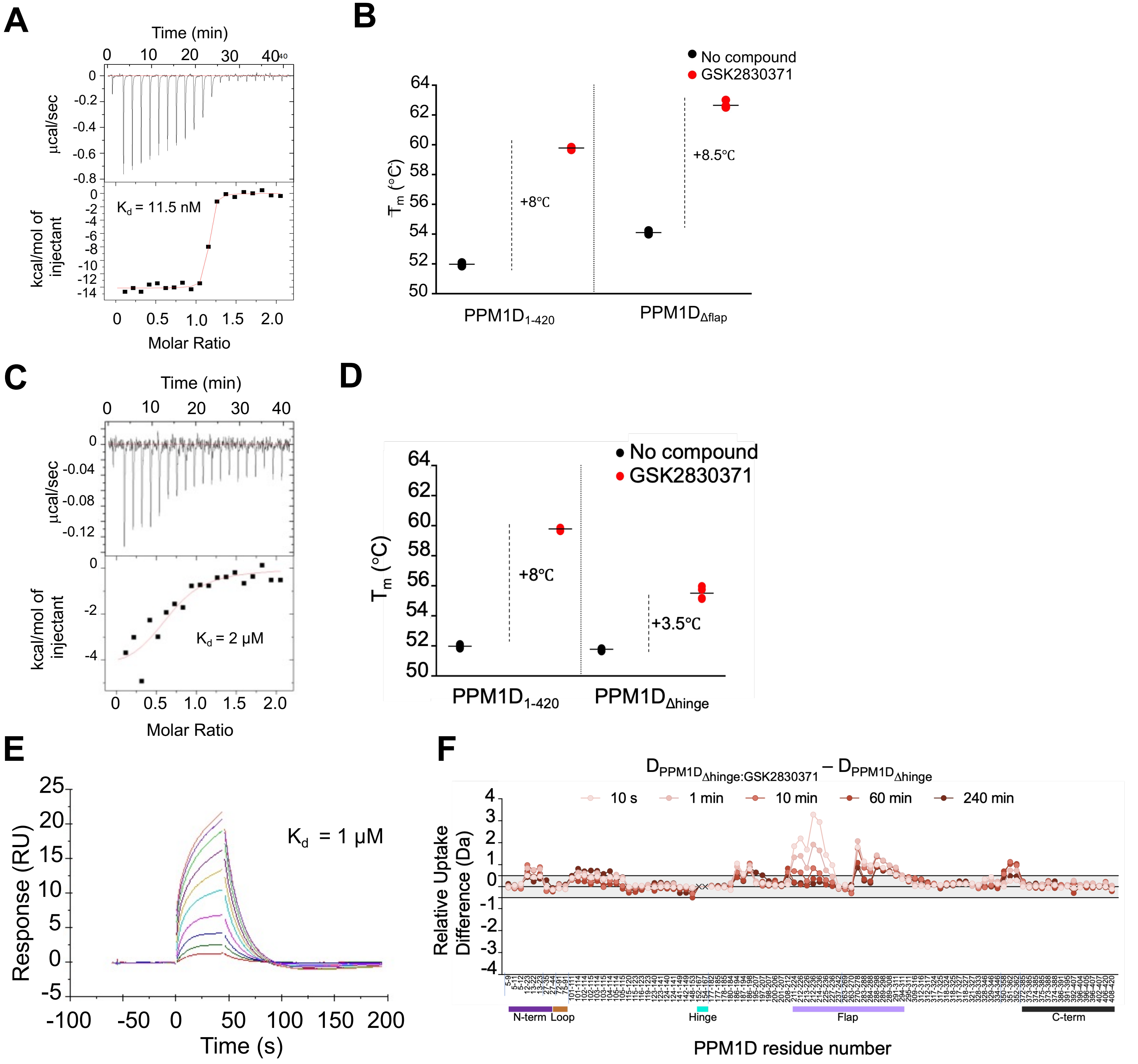

Supplementary Figure 5

(A) ITC of binding between $PPM1D_{\Delta flap}$ and GSK2830371. N=2 independent sample runs. Data are presented as mean values +/- SD.

(B) Differential scanning calorimetry (DSC) of PPM1D1-420 and PPM1DΔflap in the absence (black) or presence (red) of GSK2830371. N=4 independent sample runs. Data are presented as mean values +/- SD.

(C) DSC of GSK2830371 binding to $PPM1D_{\Delta hinge}$. N=2 independent sample runs. Data are presented as mean values +/- SD.

(D) ITC of binding between $PPM1D_{\Delta hinge}$ and GSK2830371. N=4 independent sample runs. The horizontal bars represent mean values.

(E) SPR of binding between $PPM1D_{\Delta hinge}$ and GSK2830371.

(F) Relative differences in deuterium uptake between $PPM1D_{\Delta hinge}$ in the presence of GSK2830371 compared to $PPM1D_{\Delta hinge}$ without GSK2830371 as assessed by HDX-MS. Vertical tick marks between peptides on the X-axis are shown to indicate regions of missing coverage. The bars below the x-axis represent the N-terminus (purple), loop (brown), hinge (cyan), flap (light purple), and C-terminus (black) of the protein.

Source data are provided as a Source Data file.

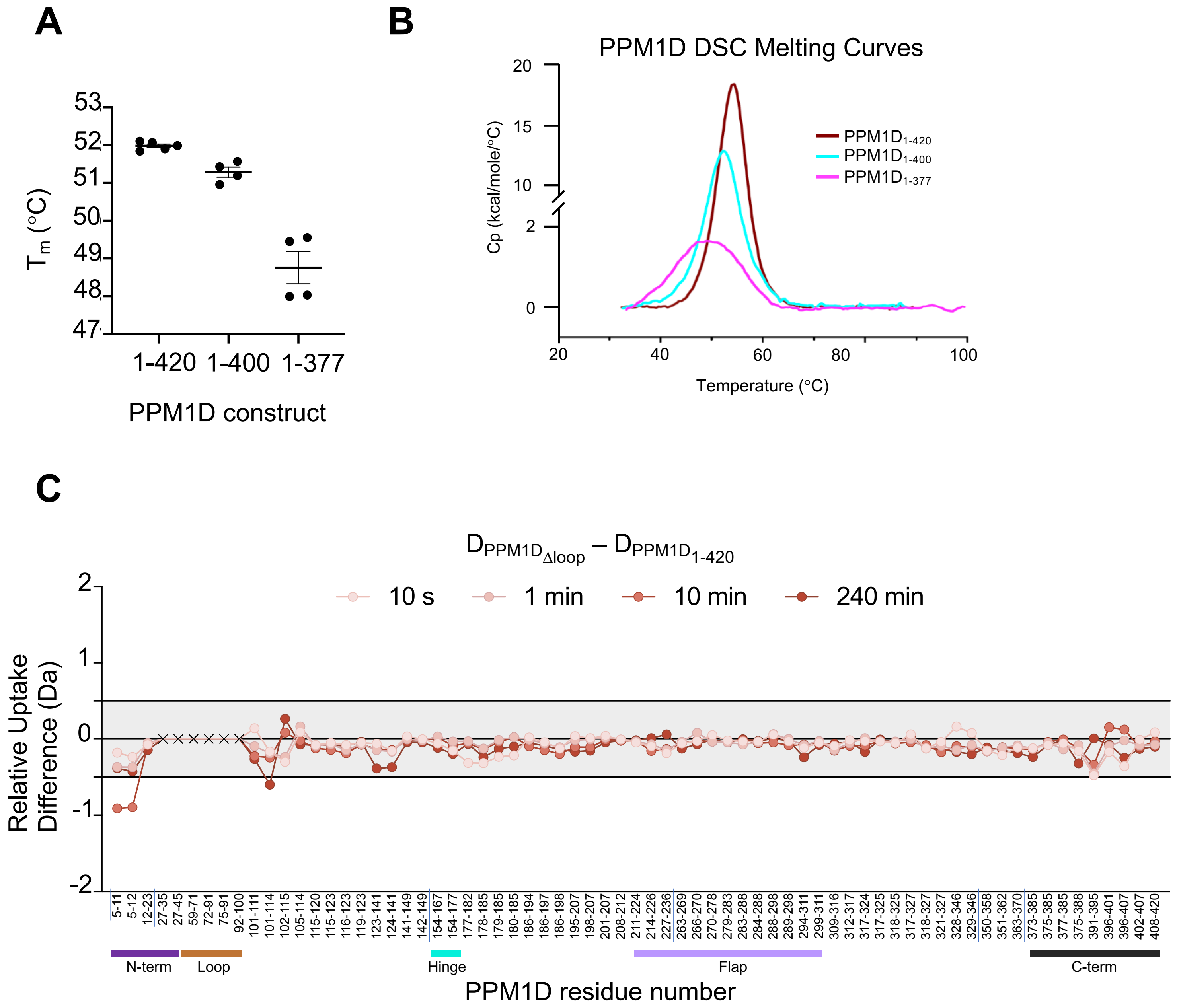

Supplementary Figure 6

(A) DSF of C-terminal truncation mutants binding to GSK2830371. N=4 independent sample runs. Data are presented as mean values +/- SD. Source data are provided as a Source Data file.

(B) Raw data for DSC shown in Figure 7B (different truncations of PPM1D).

(C) HDX-MS of $PPM1D_{\Delta 39\text{-}95}$ compared to $PPM1D_{1\text{-}420}$. Vertical tick marks between peptides on the X-axis are shown to indicate regions of missing coverage. The bars below the x-axis represent the N-terminus (purple), Loop (brown), Hinge (cyan), Flap (purple), and C-terminus (black) of the protein.

Entry	UniProtID	Protein Name	Gene Name	Sequence Length	Identity	X-Crystal
1	O15297	Protein phosphatase 1D	PPM1D	1-605	100.00%	No
2	Q8N819	Probable protein phosphatase 1N	PPM1N	1-430	12.67%	No
3	P35813	Protein phosphatase 1A	PPM1A	1-382	13.91%	Yes
4	O75688	Protein phosphatase 1B	PPM1B	1-479	15.19%	Yes
5	Q8WY54	Protein phosphatase 1E	PPM1E	1-518	9.67%	No
6	P49593	Protein phosphatase 1F	PPM1F	1-454	9.64%	No
7	Q5SGD2	Protein phosphatase 1L	PPM1L	1-360	9.89%	No
8	Q8N3J5	Protein phosphatase 1K, mitochondrial	PPM1K	1-372	10.64%	Yes
9	O15355	Protein phosphatase 1G	PPM1G	1-546	10.22%	No
10	Q9ULR3	Protein phosphatase 1H	PPM1H	1-514	7.65%	No
11	Q96MI6	Protein phosphatase 1M	PPM1M	1-270	5.40%	No
12	Q5JR12	Protein phosphatase 1J	PPM1J	1-505	8.03%	No

Supplementary Table 1A: Human PPM1D Paralog Aligned by Identity

Entry	UniProtID	Protein Name	Gene Name	Sequence Length	Identity	X-Crystal
1	O15297	Protein phosphatase 1D	PPM1D	1-605	100.00%	No
2	Q8N819	Probable protein phosphatase 1N	PPM1N	1-430	21.21%	No
3	P35813	Protein phosphatase 1A	PPM1A	1-382	21.25%	Yes
4	O75688	Protein phosphatase 1B	PPM1B	1-479	24.81%	Yes
5	Q8WY54	Protein phosphatase 1E	PPM1E	1-518	17.33%	No
6	P49593	Protein phosphatase 1F	PPM1F	1-454	16.80%	No
7	Q5SGD2	Protein phosphatase 1L	PPM1L	1-360	16.24%	No
8	Q8N3J5	Protein phosphatase 1K, mitochondrial	PPM1K	1-372	17.45%	Yes
9	O15355	Protein phosphatase 1G	PPM1G	1-546	15.63%	No
10	Q9ULR3	Protein phosphatase 1H	PPM1H	1-514	15.31%	No
11	Q96MI6	Protein phosphatase 1M	PPM1M	1-270	11.09%	No
12	Q5JR12	Protein phosphatase 1J	PPM1J	1-505	14.83%	No

Supplementary Table 1B: Human PPM1D Paralog Aligned by Similarity

Strategy	Construct	Additives	Partner protein
Wild-type	PPM1D(1-420)		
C-terminal truncation	PPM1D(1-400)		
	PPM1D(1-383)		
	PPM1D(1-377)		
Loop1 truncation	PPM1D(1-420, Δ39-60)		
	PPM1D(1-420, Δ39-70)		
	PPM1D(1-420, Δ39-80)		
	PPM1D(1-420, Δ39-95)		
Flap region truncation	PPM1D(1-246, NGS, 269-420)		
	PPM1D(1-420, Flap1)		
	PPM1D(1-420, Flap2)		
	PPM1D(1-420, Flap4)		
	PPM1D(1-420, Flap5)		
	PPM1D(1-420, Flap6)		
MBP Fusion	MBP-PPM1D(1-420)	maltose	
	MBP-AAAA-PPM1D(2-420)	maltose	
Minimal constructs	PPM1D(1-400, Δ39-95)		
	PPM1D(1-400, Δ39-95, Flap2)		Fab
	PPM1D(1-400, Δ39-95, Flap5)		
	PPM1D(1-400, Δ39-60)		
	PPM1D(1-400, Δ39-60, Flap6)		Fab
	PPM1D(1-400, Δ39-95, Flap6)		Fab
	PPM1D(1-400, Δ39-60, Flap7)		Fab
	PPM1D(1-400, Δ39-95, Flap7)		Fab
T4L fusion	PPM1D(1-400, Δ39-60, Flap7-T4L)		
	PPM1D(1-400, Δ39-95, Flap7-T4L)		
	PPM1D(1-420, Δ48-80-T4L)		
	PPM1D(1-420, Flap5-T4L)		
	PPM1D(1-420, Δ61-69-T4L)		
Combo-strategy	MBP-PPM1D(1-400, Δ39-95, Flap2)	maltose	
	MBP-AAAA-PPM1D(1-400, Δ39-95, Flap2)	maltose	
	PPM1D(1-400, Δ48-80-T4L, Flap2)		
	PPM1D(1-400, Δ41-80-T4L, Flap2)		
	PPM1D(1-400, Δ48-93-T4L, Flap2)		
	PPM1D(1-400, Δ41-93-T4L, Flap2)		
	MBP-PPM1D(2-400, Δ48-80-T4L, Flap2)	maltose	
	MBP-AAAA-PPM1D(1-400, Δ48-80-T4L, Flap2)	maltose	
Other species	Lizard PPM1D(1-326)		
	Zebrafish PPM1D(1-377)		
Metal-mediated T4L oligomerization	PPM1D(1-400, Δ39-95, Flap7-T4L-R76H, R80H)	Cu2+	T4L-D61H, K65H, R76H, R80H
SER	PPM1D(1-400, Δ39-95, Flap2, SER123)		

Supplementary Table 2: Strategies Attempted for PPM1D Crystalizatio

Entry	Identity	Organism	Gene names
O15297	100.00%	Homo sapiens (Human)	PPM1D WIP1
H2NUD3	98.70%	Pongo abelii (Sumatran orangutan) (Pongo pygmaeus abelii)	PPM1D
I0FVT7	98.50%	Macaca mulatta (Rhesus macaque)	PPM1D
G3R0Z4	98.50%	Gorilla gorilla gorilla (Western lowland gorilla)	
F7IIZ4	96.90%	Callithrix jacchus (White-tufted-ear marmoset)	PPM1D
H0WHW0	94.70%	Otolemur garnettii (Small-eared galago) (Garnett's greater bushbaby)	PPM1D
F1PFI9	94.40%	Canis lupus familiaris (Dog) (Canis familiaris)	PPM1D
I3LH52	93.90%	Sus scrofa (Pig)	PPM1D
I3N4Y5	93.60%	Ictidomys tridecemlineatus (Thirteen-lined ground squirrel) (Spermophilus tridecemlineatus)	PPM1D
G3SYF7	93.40%	Loxodonta africana (African elephant)	PPM1D
F7D2H3	92.40%	Equus caballus (Horse)	PPM1D
H0UTQ0	91.90%	Cavia porcellus (Guinea pig)	PPM1D
E1BD03	91.90%	Bos taurus (Bovine)	PPM1D
G1NTE0	91.70%	Myotis lucifugus (Little brown bat)	PPM1D
B1WCA0	89.30%	Rattus norvegicus (Rat)	Ppm1d
Q9QZ67	88.30%	Mus musculus (Mouse)	Ppm1d Wip1
G3RWQ1	98.30%	Gorilla gorilla gorilla (Western lowland gorilla)	
F7IJ28	97.60%	Callithrix jacchus (White-tufted-ear marmoset)	PPM1D
G1QHR8	86.90%	Nomascus leucogenys (Northern white-cheeked gibbon) (Hylobates leucogenys)	PPM1D

Supplementary Table 3: Orthologs Used for Evolutionary Conservation Analysis

Data Set	$PPM1D_{(1-420)}$	Hinge and flap deletions		C-terminal truncations	Loop deletion
**Related Figures**	Figures 4A, 4B, 5A, 5B	Figures 4C, 4D, 4E, 4F, 6B, 6D, Supp. 5F		Figure 7D	Supplementary Figure 6B
**States analyzed**	$PPM1D_{(1-420)}$ + DMSO<br>$PPM1D_{(1-420)}$ + GSK	+DMSO<br>$PPM1D_{(1-420)}$<br>$PPM1D_{\Delta flap}$<br>$PPM1D_{\Delta hinge}$	+GSK<br>$PPM1D_{(1-420)}$<br>$PPM1D_{\Delta flap}$<br>$PPM1D_{\Delta hinge}$	$PPM1D_{(1-420)}$<br>$PPM1D_{(1-400)}$<br>$PPM1D_{(1-377)}$	$PPM1D_{(1-420)}$<br>$PPM1D_{\Delta loop}$
**HDX reaction details (a)**	Final D_2O concentration = 93.6%, pH_{read} = 7.1				
**HDX time course**	0.167, 1, 10, 60, 240 minutes				
**Back-exchange (b)**	~35%				
**HDX controls**	3 undeuterated + DMSO	8 undeuterated; 2-3 per state + DMSO		3 undeuterated; $PPM1D_{(1-420)}$	3 undeuterated; $PPM1D_{(1-420)}$
**Number of peptides**	79 followed, 102 identified	96 followed, 148 identified		128 followed, 193 identified	69 followed, 82 identified
**Filtering parameters**	0.3 products per a.a.<br>3 consecutive products<br>8 ppm error<br>File threshold of 2	0.3 products per a.a.<br>3 consecutive products<br>10 ppm error<br>File threshold of 4		0.3 products per a.a.<br>3 consecutive products<br>9 ppm error<br>File threshold of 2	0.3 products per a.a.<br>3 consecutive products<br>10 ppm error<br>File threshold of 3
**Sequence coverage**	91.2%	79%		92.9%	86.4%
**Average peptide length**	13.6	11.3		12.8	10.6
**Redundancy**	2.8	3.26		4.21	2
**Replicates**	1-3 technical replicates for each state				
**Repeatability (c)**	+/- 0.20 relative Da				
**Significant differences (d)**	> 0.5 Da				

(a) 15-fold dilution with labeling buffer [20 mM HEPES, pD 7.5, 25 mM NaCl, 5 mM $MgCl_2$, 0.1 mM TCEP, 99.9% D_2O].
1:1 (v/v) dilution with quench buffer [0.8M guanidine hydrochloride, 0.8% formic acid, H_2O].
(b) Back exchange estimated using peptides from the loop region of PPM1D, specifically peptides covering residues 45-91.
(c) Average standard deviation across all replicate measurements for all peptides and timepoints for each state analyzed
(d) Global |ΔHX| significance threshold was calculated from experimental standard deviations to be 0.405 Da (Hageman TS and Weis DD. *Anal Chem* 91, 8008-8016, 2019).

Supplementary Table 4: HDX Data Summary and Experimental Parameters

Compound 2
1H NMR (DMSO-d6, 400 MHz)
7.97-8.01 (m, 1H), 7.49-7.57 (m, 2H), 7.04-7.05 (m, 1H), 6.59-6.63 (m, 2H), 4.67 (d, J = 5.6 Hz, 2H)

Compound 3
1H NMR (DMSO-d6, 400 MHz)
8.06-8.70 (m, 1H), 7.95-8.01 (m, 1H), 7.69-7.73 (m, 1H), 7.46-7.49 (m, 1H), 7.01 (d, J = 4.0 Hz, 1H), 6.58-6.62 (m, 2H), 4.63-4.65 (m, 2H), 4.43-4.48 (m, 1H), 3.62 (s, 3H), 3.07 (s, 2H), 1.56-1.71 (m, 7H)

Compound 4
1H NMR (DMSO-d6, 400 MHz)
8.48 (d, J = 8.4 Hz, 1H), 7.89-7.98 (m, 1H), 7.68-7.70 (m,1H), 7.45-7.49 (m, 1H), 7.00-7.01 (m, 1H), 6.58-6.62 (m, 2H), 4.64 (d, J = 5.6 Hz, 2H), 4.35-4.45 (m, 1H), 1.59-1.72 (m, 7H), 1.12-1.19 (m, 4H), 0.84-1.10 (m, 2H)

Compound 1d
1H NMR (DMSO-d6, 400 MHz)
8.37 (d, J = 8.4 Hz, 1H), 7.97 (t, J = 12.4 Hz, 2H), 7.72 (d, J = 3.6 Hz, 2H), 7.48 (t, J = 12 Hz 1H), 6.99 (d, J = 3.6 Hz, 1H), 6.51-6.61 (m, 2H), 4.63 (d, J = 6.4 Hz, 2H), 4.37-4.47 (m, 1H), 2.99-3.07 (m, 2H), 2.93 (s, 1H), 2.50-2.52 (m, 1H), 1.58-1.67 (m, 7H), 1.35-1.57 (m, 5H), 1.08-1.11 (m, 3H), 0.85-0.88 (m, 2H).

Supplementary Notes: Spectroscopic Data for Generation of Analog 1d

See Methods section “Synthesis of Analog 1d” and Supplementary Figure 2F for details of synthesis.
