# Supplementary material for: Allosteric inhibition of PPM1D serine/threonine phosphatase via an altered conformational state
Source: Nat Commun. 2022 Jun 30;13:3778. doi: 10.1038/s41467-022-30463-9 (PMC9246869; doi:10.1038/s41467-022-30463-9)
Supplement: Supplementary file 3 — Reporting Summary [file 41467_2022_30463_MOESM3_ESM.pdf]

nature portfolio

Corresponding author(s): Benjamin Ebert, MD, PhD

Last updated by author(s): Apr 11, 2022

Reporting Summary

Nature Portfolio wishes to improve the reproducibility of the work that we publish. This form provides structure for consistency and transparency in reporting. For further information on Nature Portfolio policies, see our Editorial Policies and the Editorial Policy Checklist.

Statistics

For all statistical analyses, confirm that the following items are present in the figure legend, table legend, main text, or Methods section.

n/a	Confirmed	
	x	The exact sample size (n) for each experimental group/condition, given as a discrete number and unit of measurement
	x	A statement on whether measurements were taken from distinct samples or whether the same sample was measured repeatedly
x		The statistical test(s) used AND whether they are one- or two-sided<br>*Only common tests should be described solely by name; describe more complex techniques in the Methods section.*
x		A description of all covariates tested
x		A description of any assumptions or corrections, such as tests of normality and adjustment for multiple comparisons
	x	A full description of the statistical parameters including central tendency (e.g. means) or other basic estimates (e.g. regression coefficient) AND variation (e.g. standard deviation) or associated estimates of uncertainty (e.g. confidence intervals)
x		For null hypothesis testing, the test statistic (e.g. F, t, r) with confidence intervals, effect sizes, degrees of freedom and P value noted<br>*Give P values as exact values whenever suitable.*
x		For Bayesian analysis, information on the choice of priors and Markov chain Monte Carlo settings
x		For hierarchical and complex designs, identification of the appropriate level for tests and full reporting of outcomes
x		Estimates of effect sizes (e.g. Cohen's d, Pearson's r), indicating how they were calculated

Our web collection on statistics for biologists contains articles on many of the points above.

Software and code

Policy information about availability of computer code

Data collection	All software used in the paper is outlined in the manuscript methods section. There was no custom software used. Software includes FACSDiva v8, FlowJo v10, and Clustal Omega.
Data analysis	No custom code or data analysis was used.

For manuscripts utilizing custom algorithms or software that are central to the research but not yet described in published literature, software must be made available to editors and reviewers. We strongly encourage code deposition in a community repository (e.g. GitHub). See the Nature Portfolio guidelines for submitting code & software for further information.

Data

Policy information about availability of data

All manuscripts must include a data availability statement. This statement should provide the following information, where applicable:

- Accession codes, unique identifiers, or web links for publicly available datasets
- A description of any restrictions on data availability
- For clinical datasets or third party data, please ensure that the statement adheres to our policy

The authors declare that all data supporting the findings of this study are available within the paper and its supplementary information files. MS and analysis files are accessible through the UCSD MassIVE data repository and can be downloaded through the following link (ftp://massive.ucsd.edu/MSV000088711/). BLAST was used to obtain the structures of human PPM1A (PDB ID: 3FXJ), human PPM1B (PDB ID: 2P8E), and a PPM-type phosphatase (PDB ID: 2I0O). Any additional information can be requested from the corresponding author.

March 2021

Field-specific reporting

Please select the one below that is the best fit for your research. If you are not sure, read the appropriate sections before making your selection.

- [x] Life sciences
- [ ] Behavioural & social sciences
- [ ] Ecological, evolutionary & environmental sciences

For a reference copy of the document with all sections, see nature.com/documents/nr-reporting-summary-flat.pdf

Life sciences study design

All studies must disclose on these points even when the disclosure is negative.

Sample size	Sample sizes were chosen based on the throughput of the assays, expected run-to-run variability, and examination of the data. The samples sizes selected for this study were sufficient to define differences between groups.
Data exclusions	No data exclusion was performed.
Replication	Multiple replicates of the experiments were performed including both technical and biological replicates. The details of the number of replicates are outlined in each individual figure legend. All replications were successful.
Randomization	This is not relevant to the study as there is were not analyses that required controls for covariates.
Blinding	Blinding was not relevant to this study nor was not possible as all experiments performed were designed and executed by the authors and the setup of the experiments required knowledge of the groups.

Reporting for specific materials, systems and methods

We require information from authors about some types of materials, experimental systems and methods used in many studies. Here, indicate whether each material, system or method listed is relevant to your study. If you are not sure if a list item applies to your research, read the appropriate section before selecting a response.

Materials & experimental systems

n/a	Involved in the study	
x		Antibodies
	x	Eukaryotic cell lines
x		Palaeontology and archaeology
x		Animals and other organisms
x		Human research participants
x		Clinical data
x		Dual use research of concern

Methods

n/a	Involved in the study	
x		ChIP-seq
	x	Flow cytometry
x		MRI-based neuroimaging

Eukaryotic cell lines

Policy information about cell lines

Cell line source(s)	K562 cells (ATCC)
Authentication	SNP Genotyping
Mycoplasma contamination	Tested Negative for mycoplasma
Commonly misidentified lines (See ICLAC register)	No commonly misidentified cell lines were used in this study.

Flow Cytometry

Plots

Confirm that:

- [x] The axis labels state the marker and fluorochrome used (e.g. CD4-FITC).
- [x] The axis scales are clearly visible. Include numbers along axes only for bottom left plot of group (a 'group' is an analysis of identical markers).
- [x] All plots are contour plots with outliers or pseudocolor plots.
- [x] A numerical value for number of cells or percentage (with statistics) is provided.

Methodology

Sample preparation	Cell lines were washed twice and resuspended in PBS + 5% FBS
Instrument	FACS Aria
Software	FACS Diva
Cell population abundance	>90% purity on GFP Hi (top 20%) and GFP Low (bottom 20%) populations
Gating strategy	GFP Hi (top 20%) GFP Low (top 20%)

- [x] Tick this box to confirm that a figure exemplifying the gating strategy is provided in the Supplementary Information.
