# Supplementary material for: Allosteric inhibition of PPM1D serine/threonine phosphatase via an altered conformational state
Source: Nat Commun. 2022 Jun 30;13:3778. doi: 10.1038/s41467-022-30463-9 (PMC9246869; doi:10.1038/s41467-022-30463-9)
Supplement: Supplementary file 4 — Description of Additional Supplementary Files [file 41467_2022_30463_MOESM4_ESM.docx]

Description of Supplementary files.

1) Supplementary Data 1: reference HDX Deuterium uptake plots related to figures 4A and 5A.

2) Supplementary Data 2: raw data of the HDX Deuterium uptake plots related to figures 4A and 5A.
