# Supplementary material for: Allosteric inhibition of PPM1D serine/threonine phosphatase via an altered conformational state
Source: Nat Commun. 2022 Jun 30;13:3778. doi: 10.1038/s41467-022-30463-9 (PMC9246869; doi:10.1038/s41467-022-30463-9)
Supplement: Supplementary file 5 — Supplementary Data 1 [file 41467_2022_30463_MOESM5_ESM.pdf]

PPM1D(1-420) +/- GSK2830371
Figures 4A and 5A

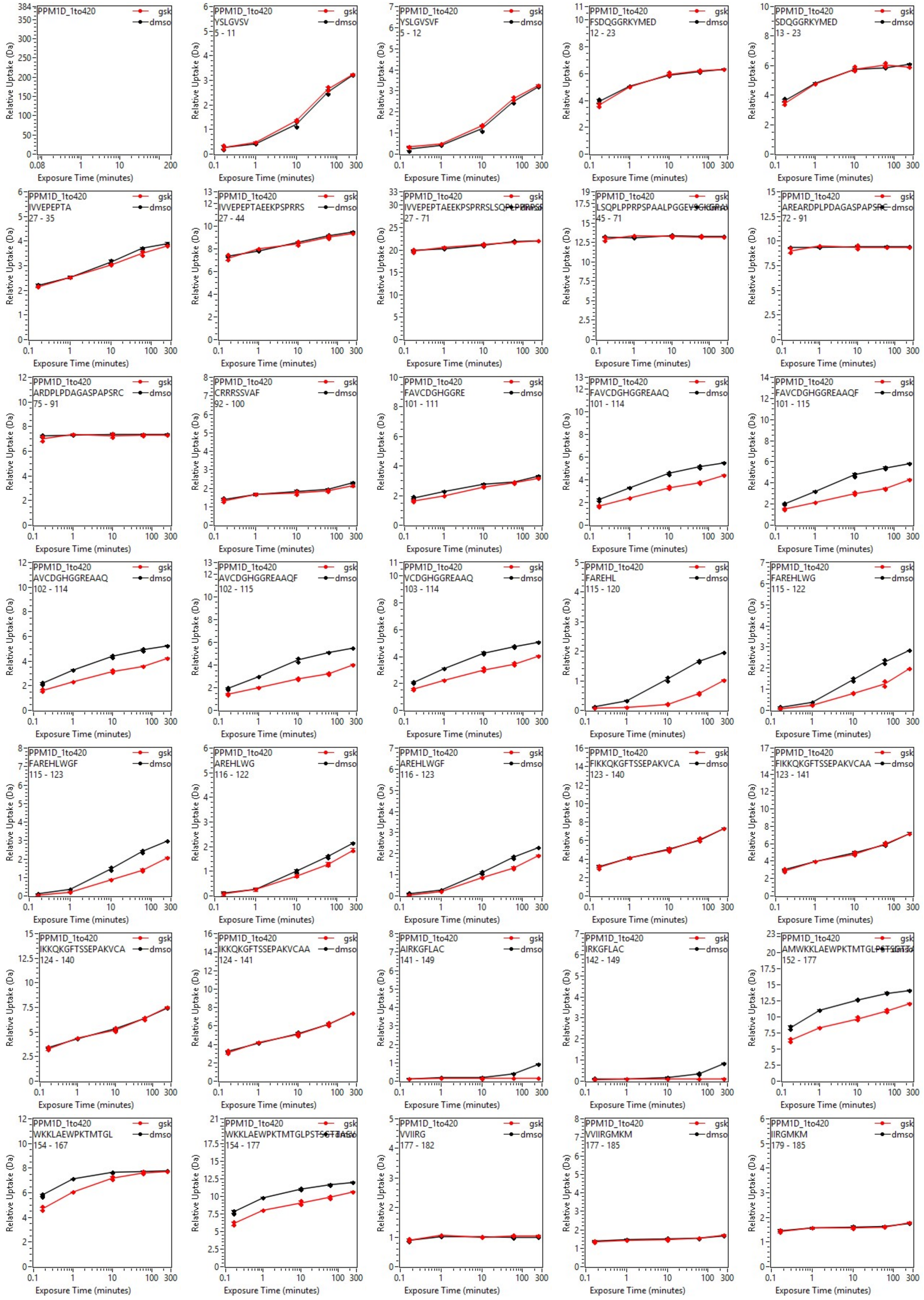

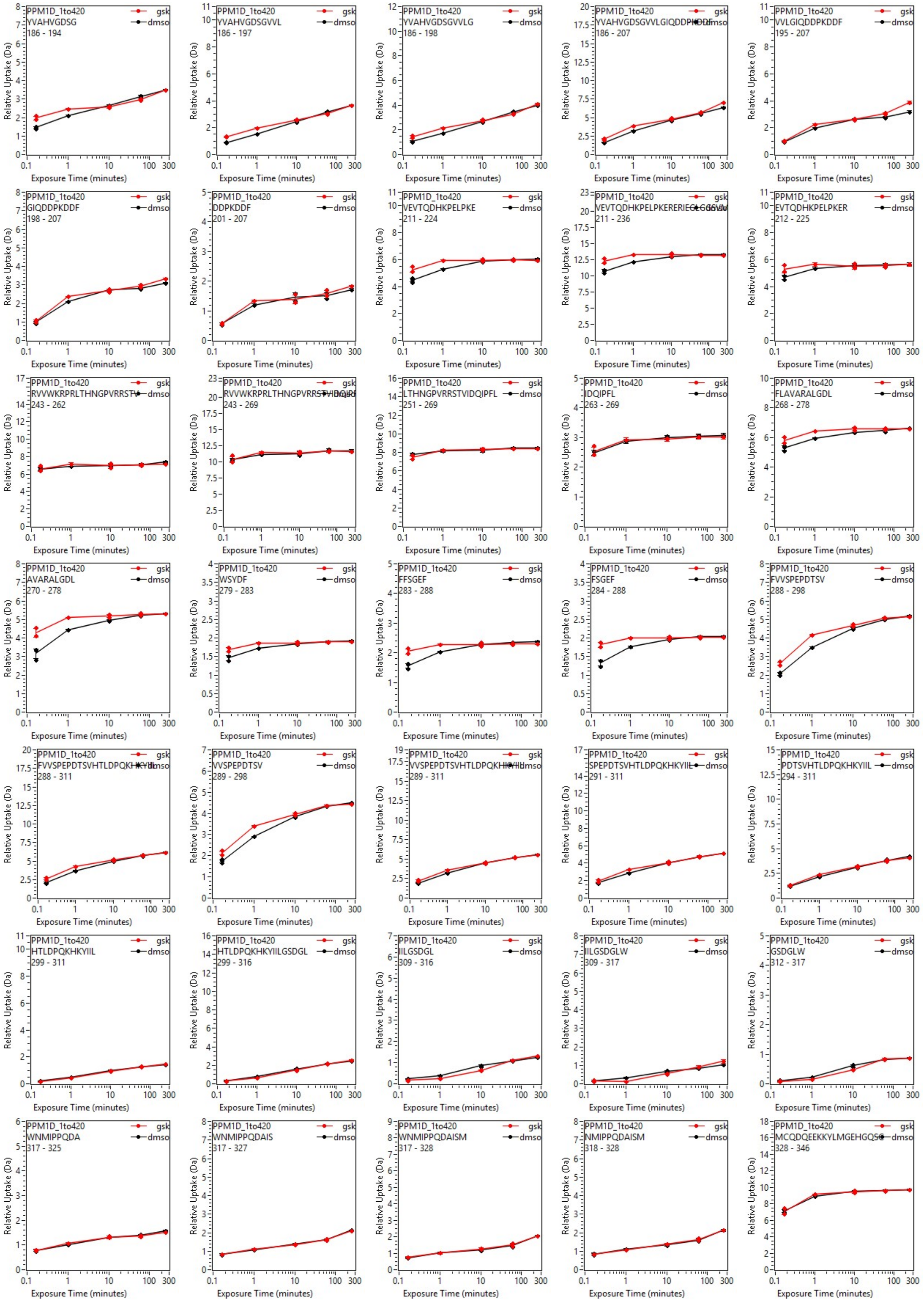
PPM1D_1to420
gsk
dmso
Relative Uptake (Da)
Exposure Time (minutes)
YVAHVGDSG
186 - 194
YVAHVGDSGVVL
186 - 197
YVAHVGDSGVVLG
186 - 198
186 - 207
VVLGIQDDPKDDF
195 - 207
GIQDDPKDDF
198 - 207
DDPKDDF
201 - 207
VEVTQDHKPELPKE
211 - 224
211 - 236
EVTQDHKPELPKER
212 - 225
243 - 262
243 - 269
LTHNGPVRRSTVIDQIPFL
251 - 269
IDQIPFL
263 - 269
FLAVARALGDL
268 - 278
AVARALGDL
270 - 278
WSYDF
279 - 283
FFSGEF
283 - 288
FSGEF
284 - 288
FVVSPEPDTSV
288 - 298
288 - 311
VVSPEPDTSV
289 - 298
289 - 311
291 - 311
PDTSVHTLDPQKHKYIIL
294 - 311
HTLDPQKHKYIIL
299 - 311
HTLDPQKHKYIILGSDGL
299 - 316
IILGSDGL
309 - 316
IILGSDGLW
309 - 317
GSDGLW
312 - 317
WNMIPPQDA
317 - 325
WNMIPPQDAIS
317 - 327
WNMIPPQDAISM
317 - 328
NMIPPQDAISM
318 - 328
328 - 346

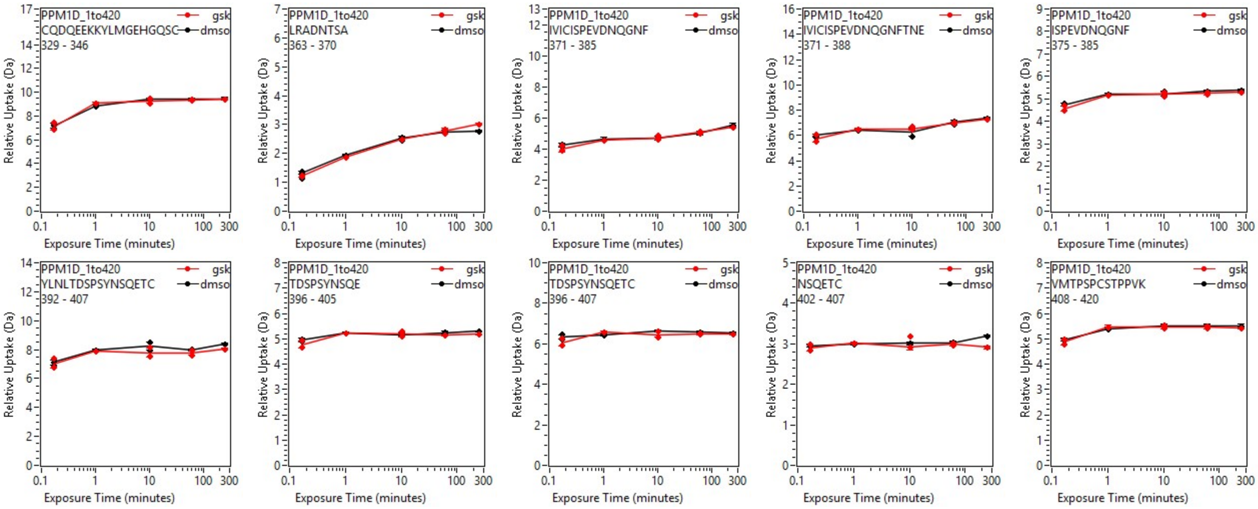

PPM1D_1to420
CQDQEEKKYLMGEHGQSC
329 - 346
LRADNTSA
363 - 370
IVICISPEVDNQGNF
371 - 385
IVICISPEVDNQGNFTNE
371 - 388
ISPEVDNQGNF
375 - 385
YLNLTDSPSYNSQETC
392 - 407
TDSPSYNSQE
396 - 405
TDSPSYNSQETC
396 - 407
NSQETC
402 - 407
VMTPSPCSTPPVK
408 - 420
gsk
dmso
Relative Uptake (Da)
Exposure Time (minutes)

PPM1D(1-420) and PPM1D(deltaflap) +/- GSK2830371
Figures 4E and 6B

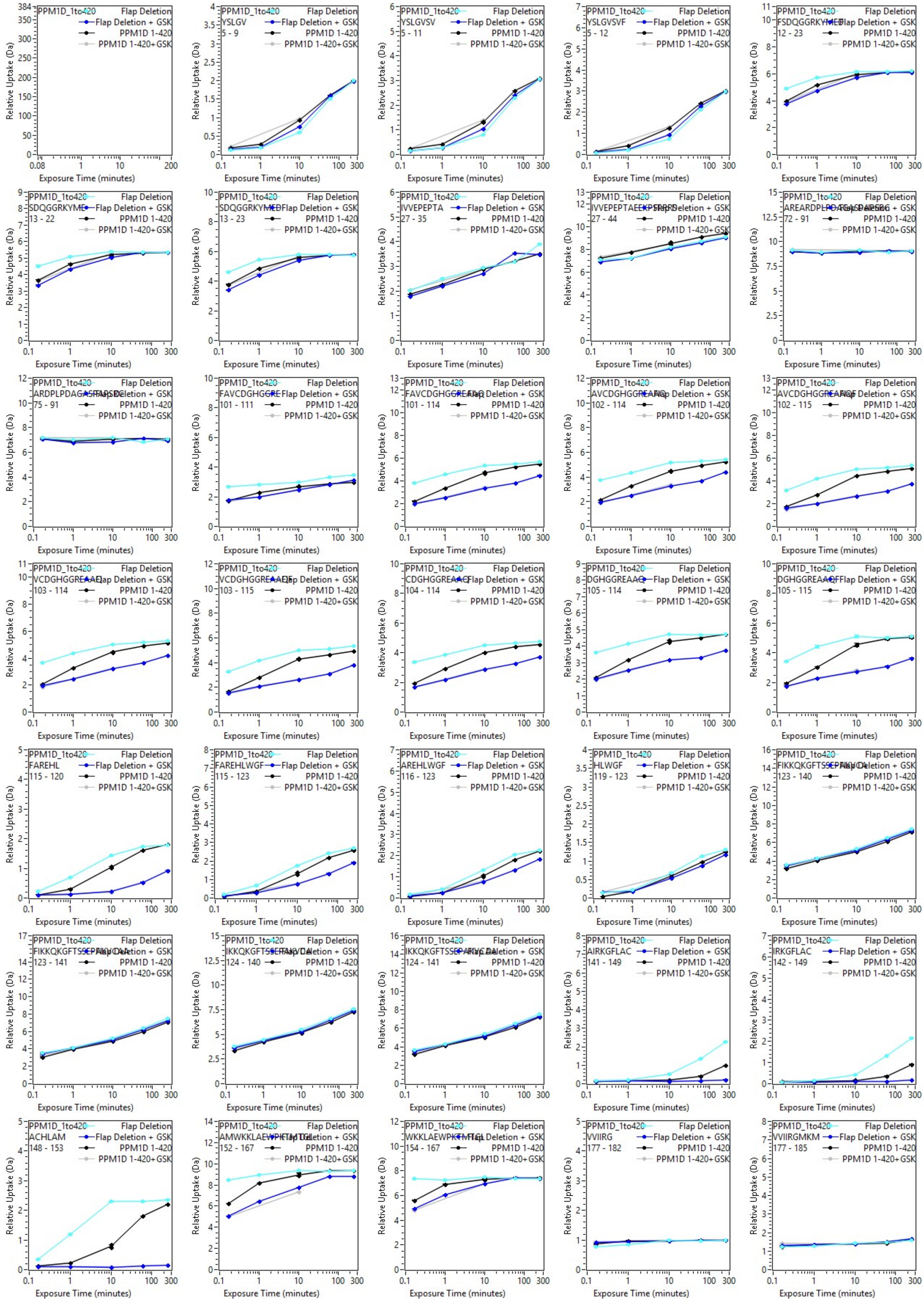

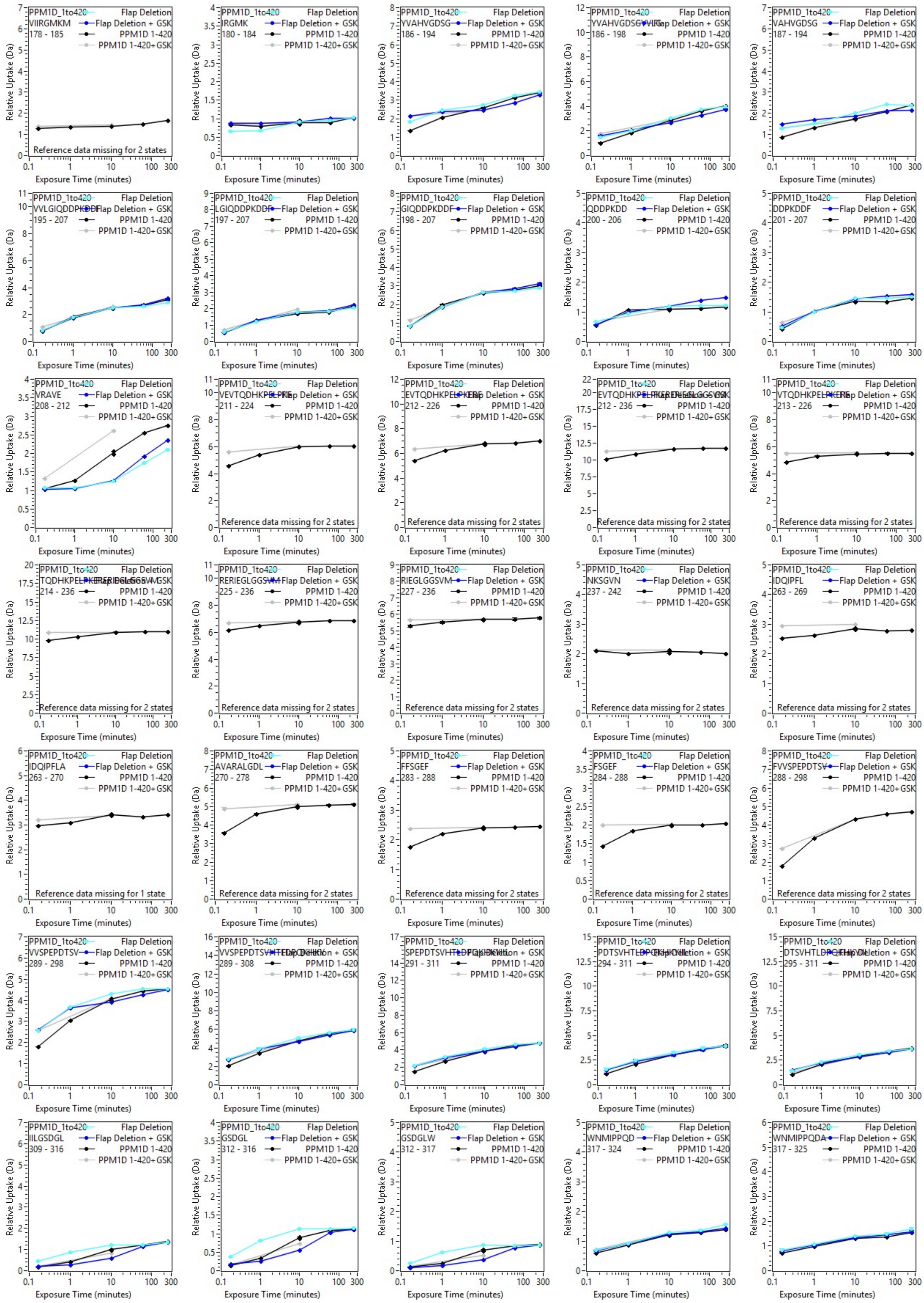
PPM1D_1to420
Flap Deletion
Flap Deletion + GSK
PPM1D 1-420
PPM1D 1-420+GSK
Relative Uptake (Da)
Exposure Time (minutes)
VIIRGMKM
178 - 185
Reference data missing for 2 states
IRGMK
180 - 184
YVAHVGDSG
186 - 194
186 - 198
VAHVGDSG
187 - 194
195 - 207
197 - 207
198 - 207
QDDPKDD
200 - 206
DDPKDDF
201 - 207
VRAVE
208 - 212
211 - 224
Reference data missing for 2 states
212 - 226
Reference data missing for 2 states
212 - 236
Reference data missing for 2 states
213 - 226
Reference data missing for 2 states
214 - 236
Reference data missing for 2 states
225 - 236
Reference data missing for 2 states
RIEGLGGSVM
227 - 236
Reference data missing for 2 states
NKSGVN
237 - 242
Reference data missing for 2 states
IDQIPFL
263 - 269
Reference data missing for 2 states
IDQIPFLA
263 - 270
Reference data missing for 1 state
AVARALGDL
270 - 278
Reference data missing for 2 states
FFSGEF
283 - 288
Reference data missing for 2 states
FSGEF
284 - 288
Reference data missing for 2 states
288 - 298
Reference data missing for 2 states
289 - 298
289 - 308
291 - 311
294 - 311
295 - 311
IILGSDGL
309 - 316
GSDGL
312 - 316
GSDGLW
312 - 317
WNMIPPQD
317 - 324
WNMIPPQDA
317 - 325

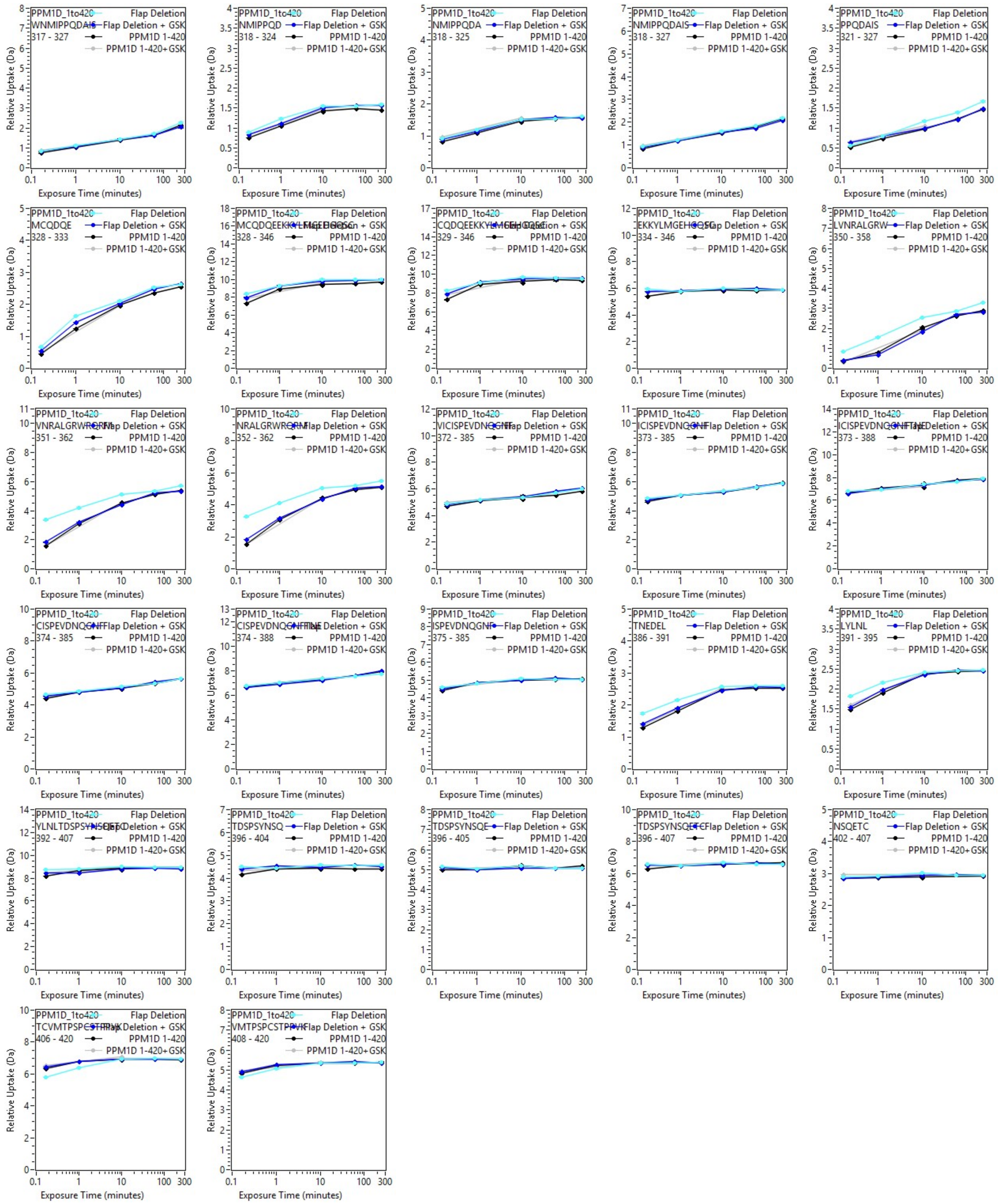
PPM1D_1to420
Flap Deletion
Flap Deletion + GSK
PPM1D 1-420
PPM1D 1-420+GSK
Relative Uptake (Da)
Exposure Time (minutes)
WNMIPPQDAIS
317 - 327
NMIPPQD
318 - 324
NMIPPQDA
318 - 325
NMIPPQDAIS
318 - 327
PPQDAIS
321 - 327
MCQDQE
328 - 333
328 - 346
329 - 346
334 - 346
LVNRALGRW
350 - 358
351 - 362
352 - 362
372 - 385
373 - 385
373 - 388
374 - 385
374 - 388
375 - 385
TNEDEL
386 - 391
LYLNL
391 - 395
392 - 407
TDSPSYNSQ
396 - 404
TDSPSYNSQE
396 - 405
396 - 407
NSQETC
402 - 407
406 - 420
408 - 420

PPM1D(1-420) and PPM1D(deltahinge) +/- GSK2830371
Figure 4D and Supplementary Figure 5F

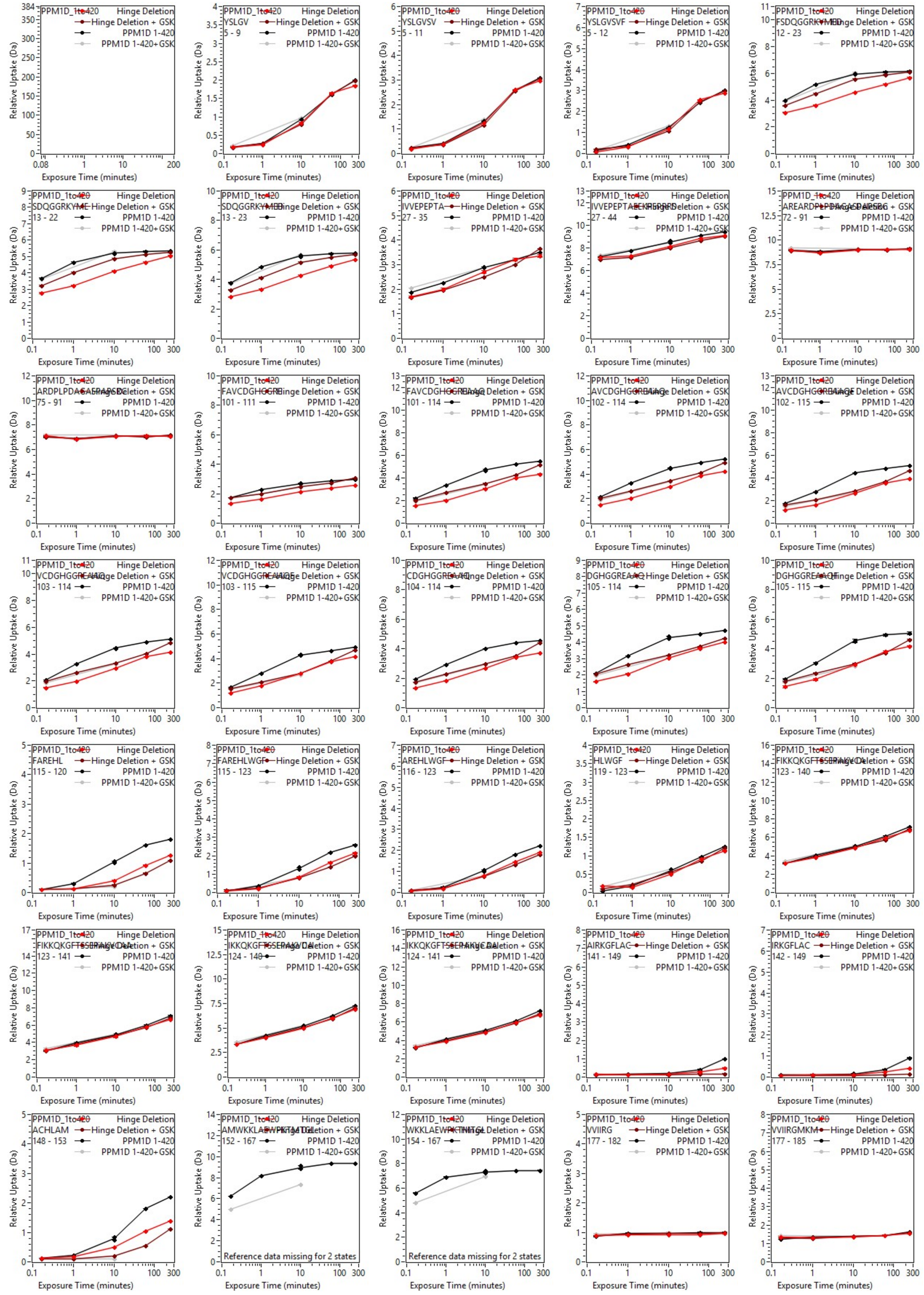

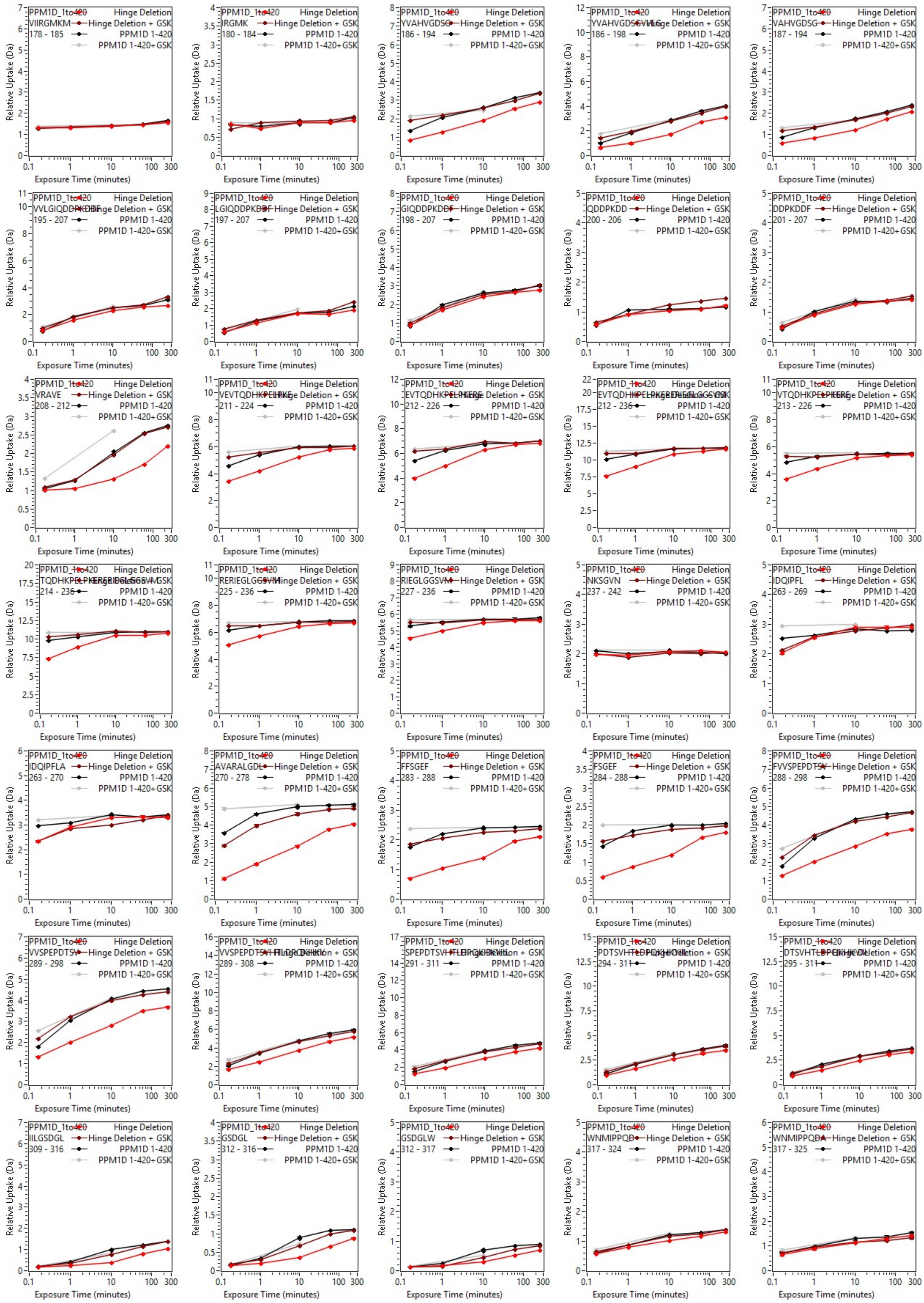
PPM1D_1to420
Hinge Deletion
Hinge Deletion + GSK
PPM1D 1-420
PPM1D 1-420+GSK
Relative Uptake (Da)
Exposure Time (minutes)
VIIRGMKM 178 - 185
IRGMK 180 - 184
YVAHVGDSG 186 - 194
186 - 198
VAHVGDSG 187 - 194
195 - 207
197 - 207
198 - 207
QDDPKDD 200 - 206
DDPKDDF 201 - 207
VRAVE 208 - 212
211 - 224
212 - 226
212 - 236
213 - 226
214 - 236
225 - 236
227 - 236
NKSGVN 237 - 242
IDQIPFL 263 - 269
IDQIPFLA 263 - 270
270 - 278
FFSGEF 283 - 288
FSGEF 284 - 288
288 - 298
289 - 298
289 - 308
291 - 311
294 - 311
295 - 311
IILGSDGL 309 - 316
GSDGL 312 - 316
GSDGLW 312 - 317
317 - 324
317 - 325

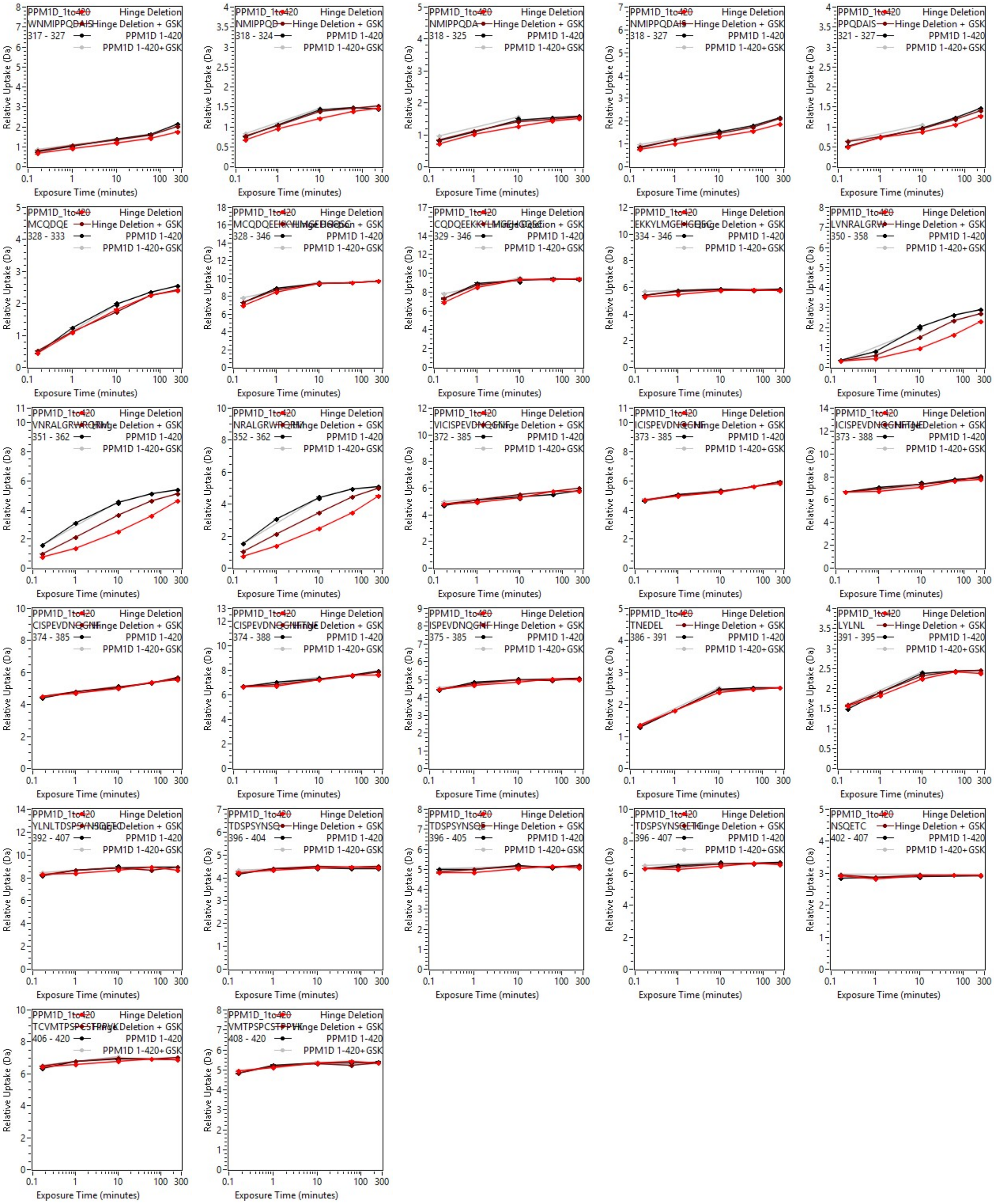

PPM1D_1to420
Hinge Deletion
Hinge Deletion + GSK
PPM1D 1-420
PPM1D 1-420+GSK
Relative Uptake (Da)
Exposure Time (minutes)
WNMIPPQDAIS 317 - 327
NMIPPQD 318 - 324
NMIPPQDA 318 - 325
NMIPPQDAIS 318 - 327
PPQDAIS 321 - 327
MCQDQE 328 - 333
328 - 346
329 - 346
334 - 346
LVNRALGRW 350 - 358
351 - 362
352 - 362
372 - 385
373 - 385
373 - 388
374 - 385
374 - 388
375 - 385
TNEDEL 386 - 391
LYLNL 391 - 395
392 - 407
TDSPSYNSQ 396 - 404
396 - 405
396 - 407
NSQETC 402 - 407
406 - 420
408 - 420

PPM1D(1-420) vs PPM1D(1-400) vs PPM1D(1-377)
Figure 7B

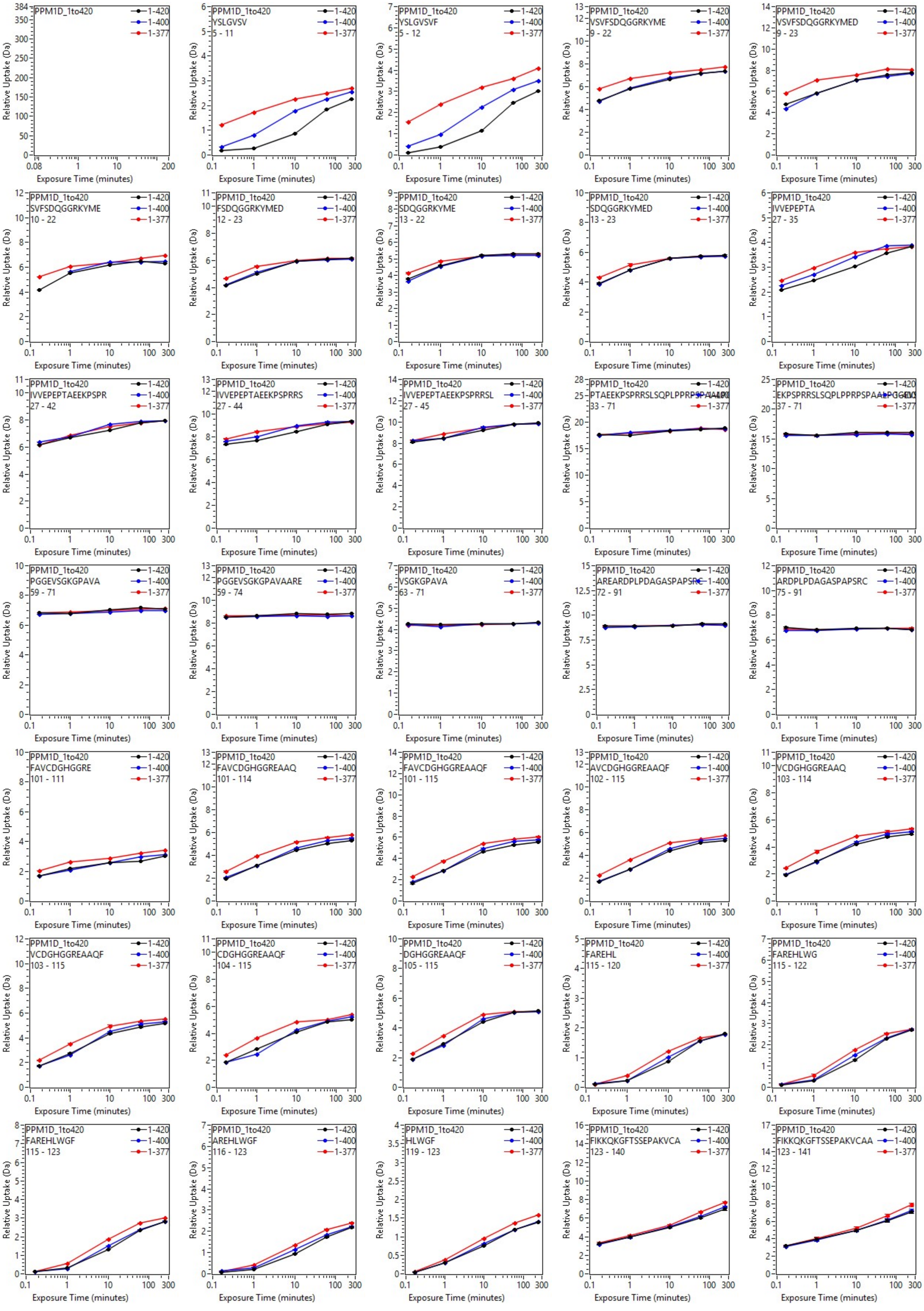

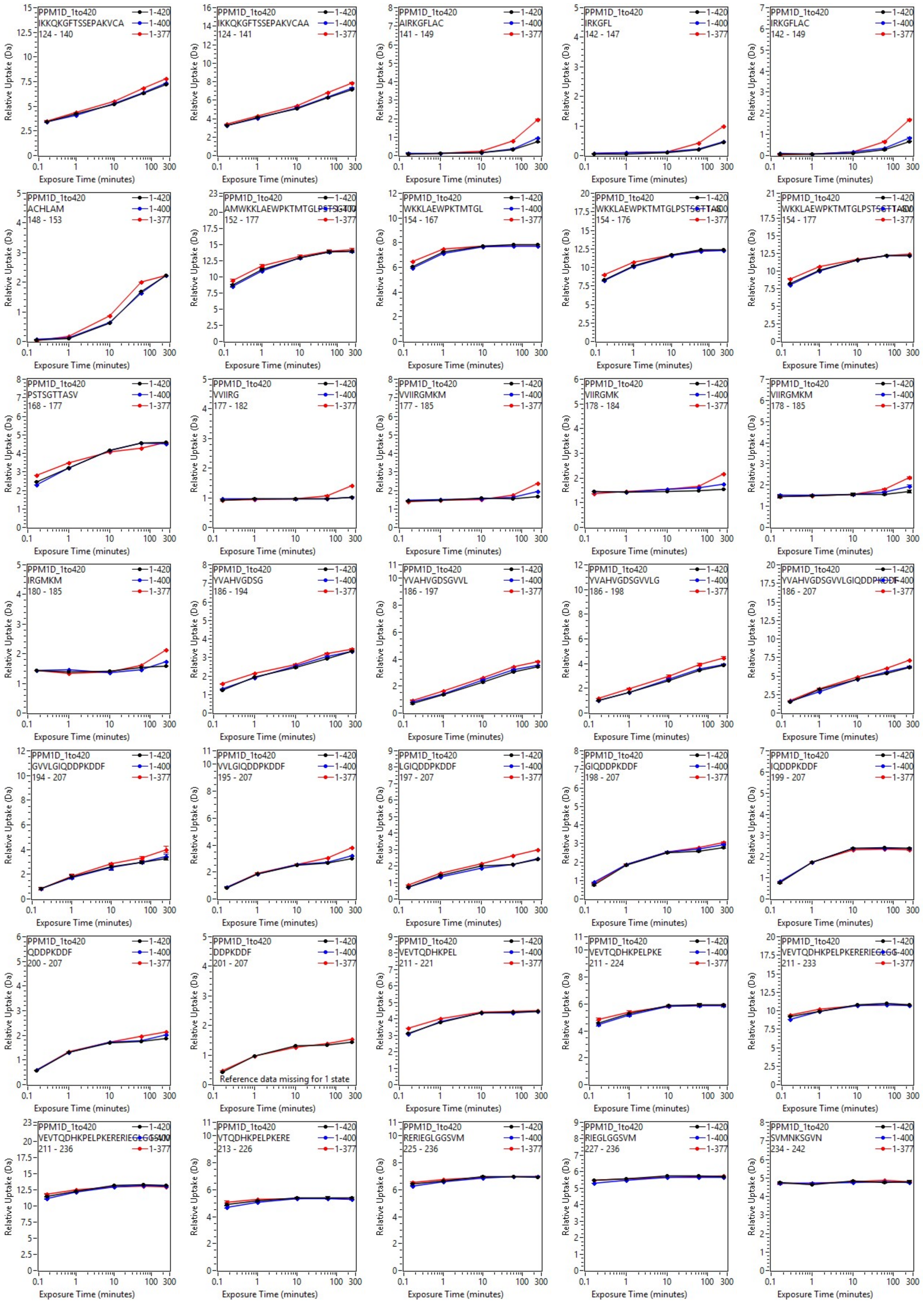
PPM1D_1to420
1-420
1-400
1-377
Relative Uptake (Da)
Exposure Time (minutes)
IKKQKGFTSSEPAKVCA
124 - 140
IKKQKGFTSSEPAKVCAA
124 - 141
AIRKGFLAC
141 - 149
IRKGFL
142 - 147
IRKGFLAC
142 - 149
ACHLAM
148 - 153
AMWKKLAEWPKTMTGLPSTSGTTASV
152 - 177
WKKLAEWPKTMTGL
154 - 167
WKKLAEWPKTMTGLPSTSGTTAS
154 - 176
WKKLAEWPKTMTGLPSTSGTTASV
154 - 177
PSTSGTTASV
168 - 177
VVIIRG
177 - 182
VVIIRGMKM
177 - 185
VIIRGMK
178 - 184
VIIRGMKM
178 - 185
IRGMKM
180 - 185
YVAHVGDSG
186 - 194
YVAHVGDSGVVL
186 - 197
YVAHVGDSGVVLG
186 - 198
YVAHVGDSGVVLGIQDDPKDDF
186 - 207
GVVLGIQDDPKDDF
194 - 207
VVLGIQDDPKDDF
195 - 207
LGIQDDPKDDF
197 - 207
GIQDDPKDDF
198 - 207
IQDDPKDDF
199 - 207
QDDPKDDF
200 - 207
DDPKDDF
201 - 207
Reference data missing for 1 state
VEVTQDHKPEL
211 - 221
VEVTQDHKPELPKE
211 - 224
VEVTQDHKPELPKERERIEGLGG
211 - 233
VEVTQDHKPELPKERERIEGLGGSVM
211 - 236
VTQDHKPELPKERE
213 - 226
RERIEGLGGSVM
225 - 236
RIEGLGGSVM
227 - 236
SVMNKSGVN
234 - 242

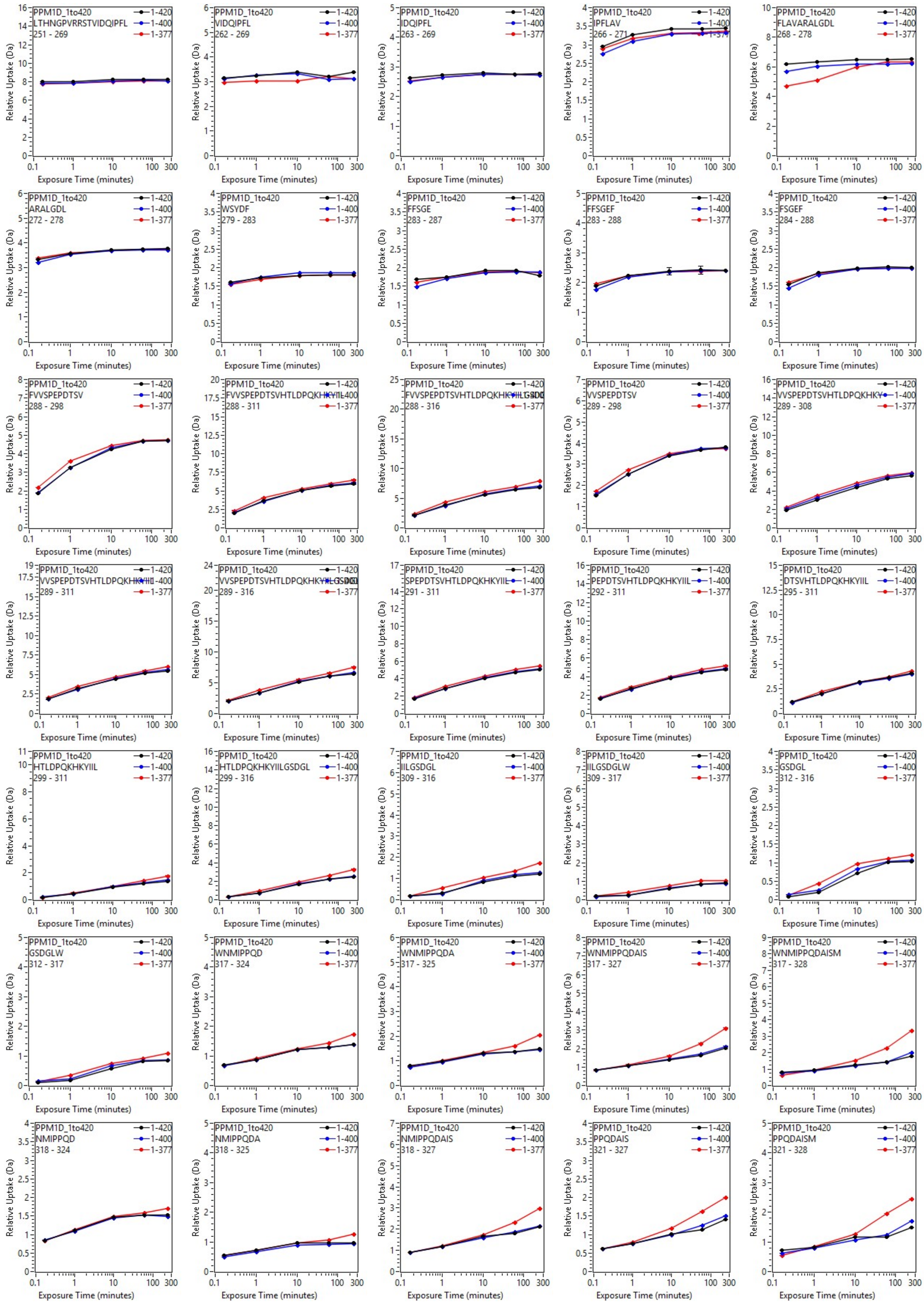

PPM1D_1to420
LTHNGPVRRSTVIDQIPFL
251 - 269
PPM1D_1to420
VIDQIPFL
262 - 269
PPM1D_1to420
IDQIPFL
263 - 269
PPM1D_1to420
IPFLAV
266 - 271
PPM1D_1to420
FLAVARALGDL
268 - 278
PPM1D_1to420
ARALGDL
272 - 278
PPM1D_1to420
WSYDF
279 - 283
PPM1D_1to420
FFSGE
283 - 287
PPM1D_1to420
FFSGEF
283 - 288
PPM1D_1to420
FSGEF
284 - 288
PPM1D_1to420
FVVSPEPDTSV
288 - 298
PPM1D_1to420
288 - 311
PPM1D_1to420
288 - 316
PPM1D_1to420
VVSPEPDTSV
289 - 298
PPM1D_1to420
289 - 308
PPM1D_1to420
289 - 311
PPM1D_1to420
289 - 316
PPM1D_1to420
SPEPDTSVHTLDPQKHKYIIL
291 - 311
PPM1D_1to420
PEPDTSVHTLDPQKHKYIIL
292 - 311
PPM1D_1to420
DTSVHTLDPQKHKYIIL
295 - 311
PPM1D_1to420
HTLDPQKHKYIIL
299 - 311
PPM1D_1to420
HTLDPQKHKYIILGSDGL
299 - 316
PPM1D_1to420
IILGSDGL
309 - 316
PPM1D_1to420
IILGSDGLW
309 - 317
PPM1D_1to420
GSDGL
312 - 316
PPM1D_1to420
GSDGLW
312 - 317
PPM1D_1to420
WNMIPPQD
317 - 324
PPM1D_1to420
WNMIPPQDA
317 - 325
PPM1D_1to420
WNMIPPQDAIS
317 - 327
PPM1D_1to420
WNMIPPQDAISM
317 - 328
PPM1D_1to420
NMIPPQD
318 - 324
PPM1D_1to420
NMIPPQDA
318 - 325
PPM1D_1to420
NMIPPQDAIS
318 - 327
PPM1D_1to420
PPQDAIS
321 - 327
PPM1D_1to420
PPQDAISM
321 - 328
1-420
1-400
1-377
Relative Uptake (Da)
Exposure Time (minutes)

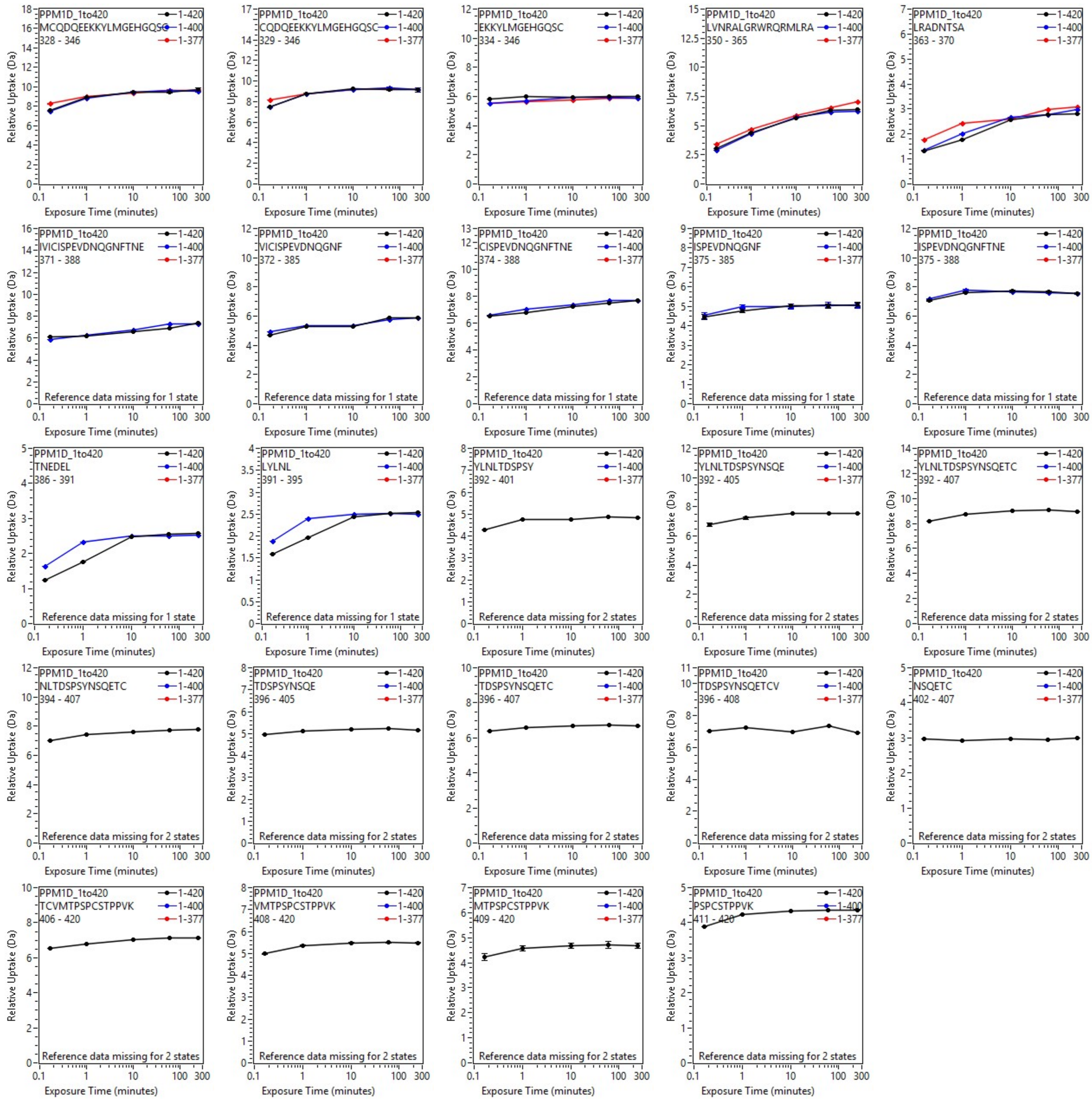
PPM1D_1to420
MCQDQEEKKYLMGEHGQSC
328 - 346
CQDQEEKKYLMGEHGQSC
329 - 346
EKKYLMGEHGQSC
334 - 346
LVNRALGRWRQRMLRA
350 - 365
LRADNTSA
363 - 370
IVICISPEVDNQGNFTNE
371 - 388
VICISPEVDNQGNF
372 - 385
CISPEVDNQGNFTNE
374 - 388
ISPEVDNQGNF
375 - 385
ISPEVDNQGNFTNE
375 - 388
TNEDEL
386 - 391
LYLNL
391 - 395
YLNLTDSPSY
392 - 401
YLNLTDSPSYNSQE
392 - 405
YLNLTDSPSYNSQETC
392 - 407
NLTDSPSYNSQETC
394 - 407
TDSPSYNSQE
396 - 405
TDSPSYNSQETC
396 - 407
TDSPSYNSQETCV
396 - 408
NSQETC
402 - 407
TCVMTPSPCSTPPVK
406 - 420
VMTPSPCSTPPVK
408 - 420
MTPSPCSTPPVK
409 - 420
PSPCSTPPVK
411 - 420
1-420
1-400
1-377
Reference data missing for 1 state
Reference data missing for 2 states
Relative Uptake (Da)
Exposure Time (minutes)
0.1
1
10
100
300

PPM1D(1-420) and PPM1D(deltaloop) +/- GSK2830371
Supplementary Figure 6B

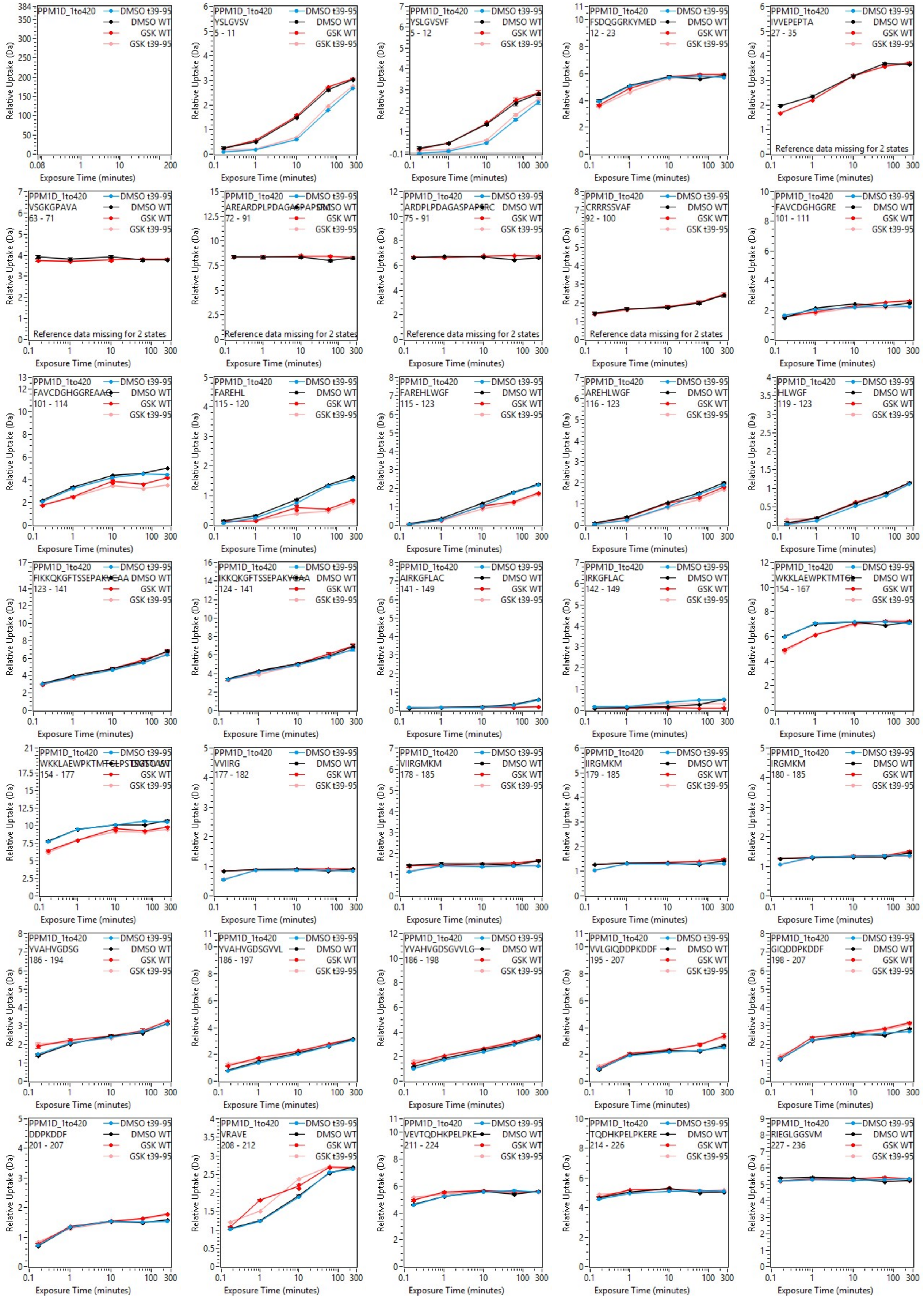

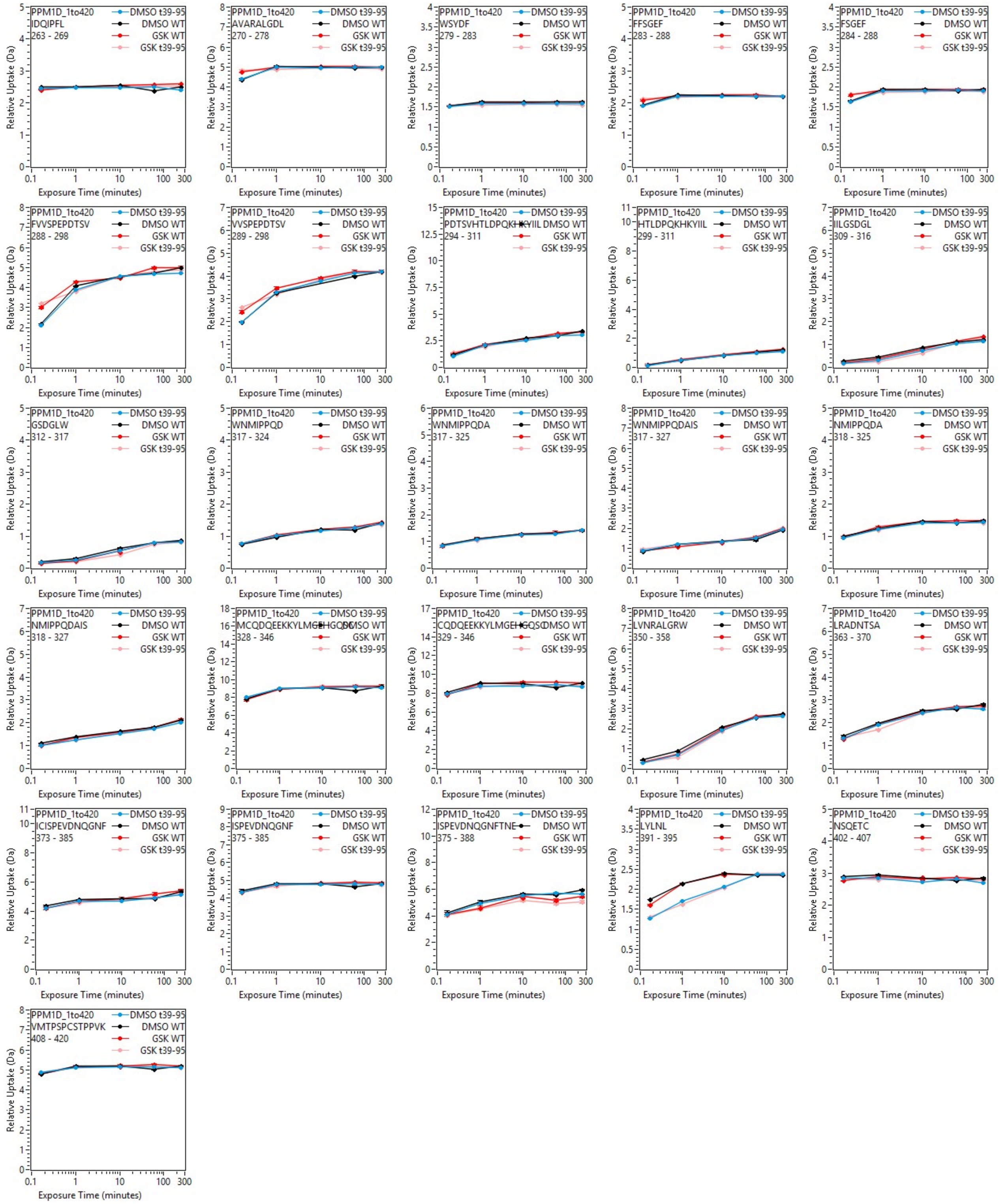
PPM1D_1to420
DMSO t39-95
DMSO WT
GSK WT
GSK t39-95
Relative Uptake (Da)
Exposure Time (minutes)
IDQIPFL 263 - 269
AVARALGDL 270 - 278
WSYDF 279 - 283
FFSGEF 283 - 288
FSGEF 284 - 288
FVVSPEPDTSV 288 - 298
VVSPEPDTSV 289 - 298
PDTSVHTLDPQKHKYIIL 294 - 311
HTLDPQKHKYIIL 299 - 311
IILGSDGL 309 - 316
GSDGLW 312 - 317
WNMIPPQD 317 - 324
WNMIPPQDA 317 - 325
WNMIPPQDAIS 317 - 327
NMIPPQDA 318 - 325
NMIPPQDAIS 318 - 327
MCQDQEEKKYLMGEHGQSC 328 - 346
CQDQEEKKYLMGEHGQSC 329 - 346
LVNRALGRW 350 - 358
LRADNTSA 363 - 370
ICISPEVDNQGNF 373 - 385
ISPEVDNQGNF 375 - 385
ISPEVDNQGNFTNE 375 - 388
LYLNL 391 - 395
NSQETC 402 - 407
VMTPSPCSTPPVK 408 - 420
